# Supplementary material for: Deposition temperature-mediated growth of helically shaped polymers and chevron-type graphene nanoribbons from a fluorinated precursor
Source: Commun Chem. 2024 Aug 31;7:193. doi: 10.1038/s42004-024-01253-9 (PMC11366011; doi:10.1038/s42004-024-01253-9)
Supplement: Supplementary file 2 — Supplementary Information [file 42004_2024_1253_MOESM2_ESM.pdf]

Deposition temperature-mediated growth of helically shaped polymers and chevron-type graphene nanoribbons from a fluorinated precursor

Jacob D. Teeter[1†], Mamun Sarker[2†], Wenchang Lu[3], Chenggang Tao[1,4], Arthur P. Baddorf[1], Jingsong Huang[1*], Kunlun Hong[1], Jerry Bernholc[3], Alexander Sinitskii[2], An-Ping Li[1,4*]

[1]Center for Nanophase Materials Sciences, Oak Ridge National Laboratory, Oak Ridge, Tennessee 37831, USA

[2]Department of Chemistry, University of Nebraska-Lincoln, Lincoln, Nebraska 68588, USA

[3]Department of Physics, North Carolina State University, Raleigh, North Carolina 27695, USA

[4]Department of Physics and Astronomy, University of Tennessee, Knoxville, Tennessee 37996, USA

Supplementary Information

Table of contents

1. **Supplementary Methods: Synthesis of precursor 1** ······ S2
2. **^{1}H, ^{13}C and ^{19}F nuclear magnetic resonance (NMR) spectra** ······ S5
3. **Additional experimental and simulated scanning tunneling microscopy (STM) images** ······ S12
4. **Additional XPS data** ······ S17

1. Supplementary Methods: Synthesis of precursor 1

General: All starting materials and reagents were purchased from commercial sources and used without further purification unless noted otherwise. The dry solvent tetrahydrofuran (THF) was freshly distilled before use. ^{1}H NMR, ^{13}C NMR, and ^{19}F NMR were recorded using Bruker 400 MHz spectrometers. Chemical shifts for ^{1}H and ^{13}C NMR are stated in parts per million (ppm, δ), downfield from tetramethylsilane (TMS, δ=0.00 ppm) and are referenced to residual solvent ($CDCl_3$, δ = 7.26 ppm (^{1}H) and 77.00 ppm (^{13}C). For ^{19}F NMR, chemical shifts (δ in ppm) are given from $CFCl_3$ as an external reference (one drop of $CFCl_3$ in $CDCl_3$ (0.00)).

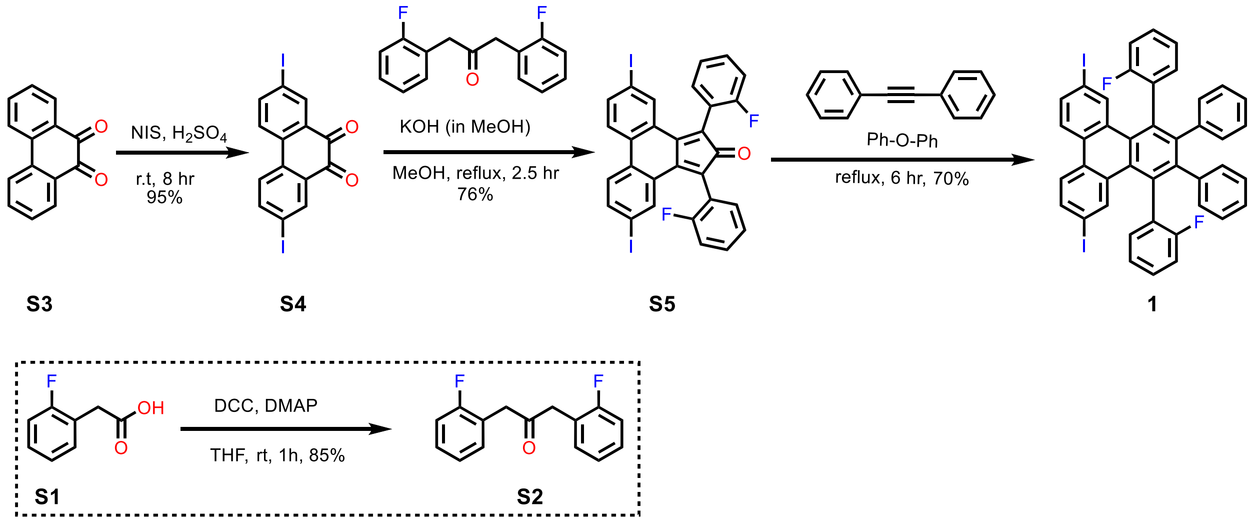

Scheme S1. Reaction scheme for the synthesis of precursor **1.**

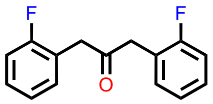

S2

<u>1,3-bis(2-fluorophenyl)propan-2-one (**S2**)</u>: 4.33 g (21.0 mmol) N,N′-dicyclohexyl-carbodiimide (DCC) and 733.02 mg (6.0 mmol) 4-dimethylaminopyridine (DMAP)

were added to stirred 18 mL dry THF in a sealed round bottom flask under nitrogen. Next, 3.08 g (20.0 mmol) 2-(2-fluorophenyl)acetic acid (**S1**) was added to the reaction mixture followed by continuous stirring for 1 hour at the room temperature. The reaction mixture was then filtered through Celite, washed with ethyl acetate, and dried under vacuum. The crude product was subsequently purified with flash column chromatography with 25% ethyl acetate in hexane as eluent, resulting in the isolation of **S2** as white crystals (4.18 g, 85% yield). **^{1}H-NMR (400 MHz, $CDCl_3$) δ:** 7.29-7.23 (m, 2H, Ar-H), 7.19-7.15 (m, 2H, Ar-H), 7.12-704 (m, 4H, Ar-H), and 3.81 (s, 4H, CH_2-H) ppm. **^{13}C-NMR (100 MHz, $CDCl_3$) δ:** 131.68, 131.64, 129.12, 129.04, 124.24, 124.20, 115.50, 115.28, and 42.34 (CH_2-C) ppm.

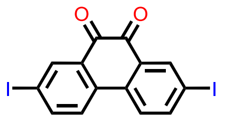

S4

2,7-diiodophenanthrene-9,10-dione (**S4**): 4.76 g (22.87 mmol) of phenanthrene-9,10-dione (**S3**) was added to 170 mL of 98% H_2SO_4 under constant stirring. Next, 18.0 g (80.05 mmol) of N-iodosuccinimide (NIS) was added to the reaction mixture below room temperature and stirred overnight. The reaction mixture was then added to an ice-water bath and filtered to yield dark orange solid of **S4** (10 g, 95% yield). **^{1}H-NMR (400 MHz, $CDCl_3$) δ:** 8.5 (d, 2H, Ar-H), 8.05-8.03 (dd, 2H, Ar-H), and 7.70 (d, 2H, Ar-H) ppm.

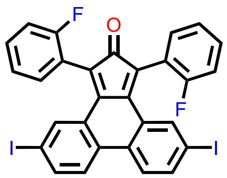

S5

1,3-bis(2-fluorophenyl)-5,10-diiodo-2H-cyclopenta[l]phenanthren-2-one (**S5**): 1428.83 mg (3.04 mmol) of **S4** and 786.06 mg (3.19 mmol) of **S2** were added to 20 mL of methanol

under constant stirring followed by heating the reaction mixture to reflux. Next, 272.87 mg (4.86 mmol) of KOH was dissolved in 5 mL of methanol and added dropwise to the reaction mixture followed by refluxing for 1 hour. The crude product appeared as green precipitate and was then filtered and washed with cold methanol and hexane to obtain isolated **S5** (1.58 g, 76% yield). **^{1}H-NMR (400 MHz, $CDCl_3$) δ:** 7.65 (d, 2H, Ar-H), 7.48-7.40 (m, 6H, Ar-H), 7.33-7.30 (m, 4H, Ar-H), and 7.20 (t, 2H, Ar-H) ppm.

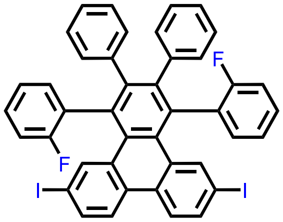

1

6,11-diiodo-1,4-bis(2-fluorophenyl)-2,3-diphenyltriphenylene (**1**): 1072.38 mg (1.6 mmol) of **S5** and 299.42 mg (1.68 mmol) of 1,3-diphenylacetylene were added in 0.5 mL di-phenyl ether in a round bottom flask. Next, the reaction mixture was refluxed for 6 hours. After cooling down to room temperature, the crude product was precipitated out by adding cold hexane and filtered. The crude product was then purified using flash chromatography resulting in **1** as white powder (918.91 mg, 70% yield). **^{1}H-NMR (400 MHz, $CDCl_3$) δ:** 8.10 (d, 2H, Ar-H), 8.02 (S, 2H, Ar-H), 7.72-7.69 (dd, 2H, Ar-H), 7.24-7.19 (m, 2H, Ar-H), 7.10-6.97 (m, 4H, Ar-H), 6.92-6.88 (m, 8H, Ar-H), 6.80-6.76 (m, 2H, Ar-H), and 6.71-6.66 (m, 2H, Ar-H) ppm. **^{13}C-NMR (100 MHz, $CDCl_3$) δ:** 137.74, 135.49, 133.10, 132.0, 131.06, 130.90, 129.62, 126.89, 126.38, 126.28, 125.78, 124.52, and 92.10 ppm. **^{19}F-NMR (376 MHz, $CDCl_3$) δ:** -111.66 - -111.71 (septet, 1F, Ar-F), -112.13 - -112.19 (septet, 1F, Ar-F).

2. ^{1}H, ^{13}C, and ^{19}F nuclear magnetic resonance (NMR) spectra

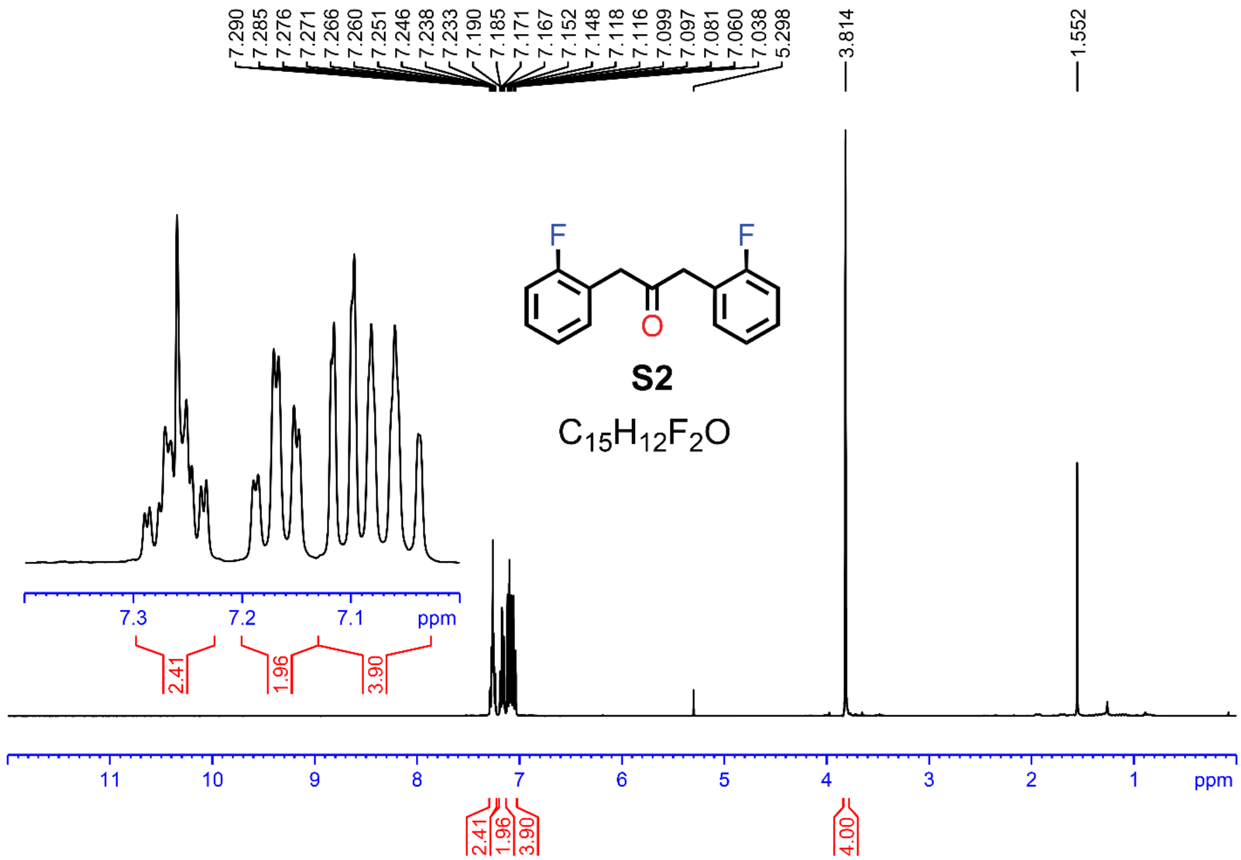

Supplementary Figure 1. ^{1}H NMR of compound **S2**.

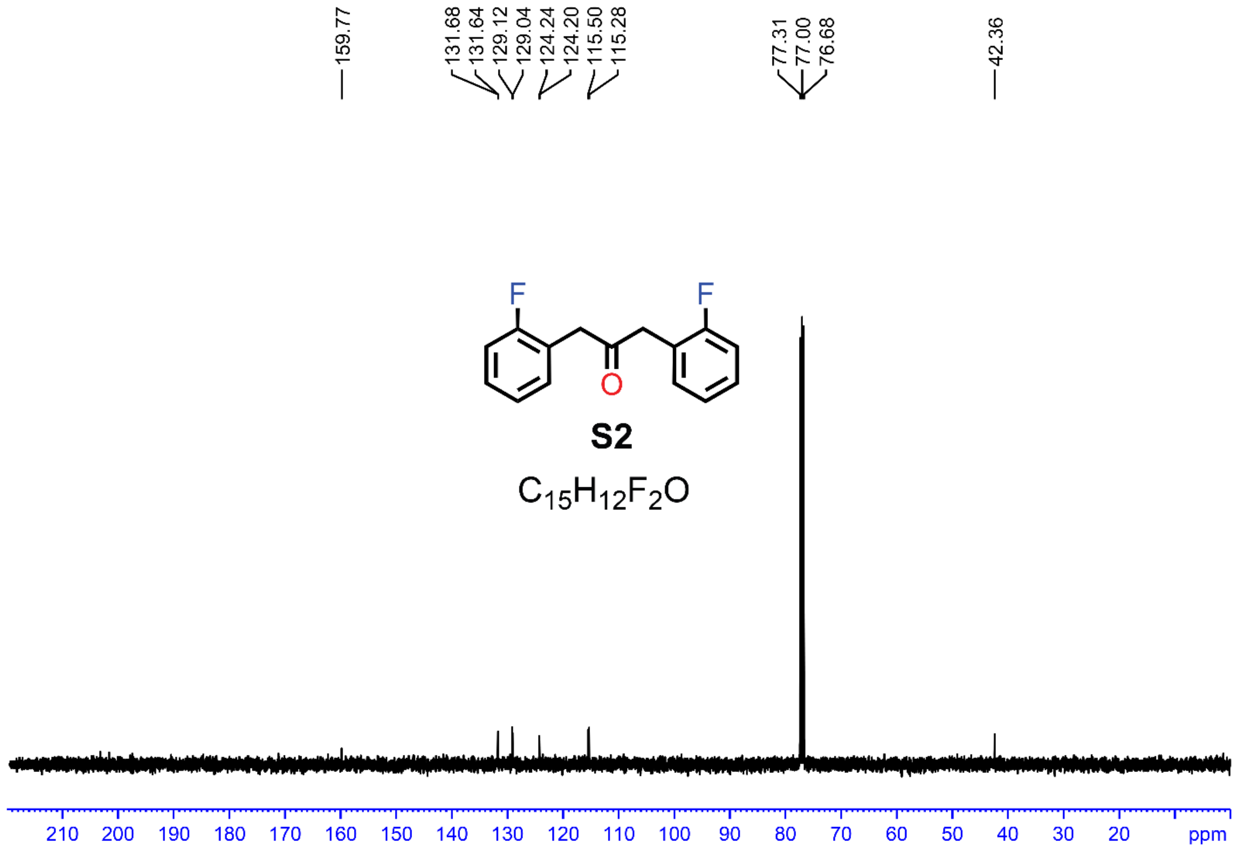

Supplementary Figure 2. ^{13}C NMR of compound **S2**.

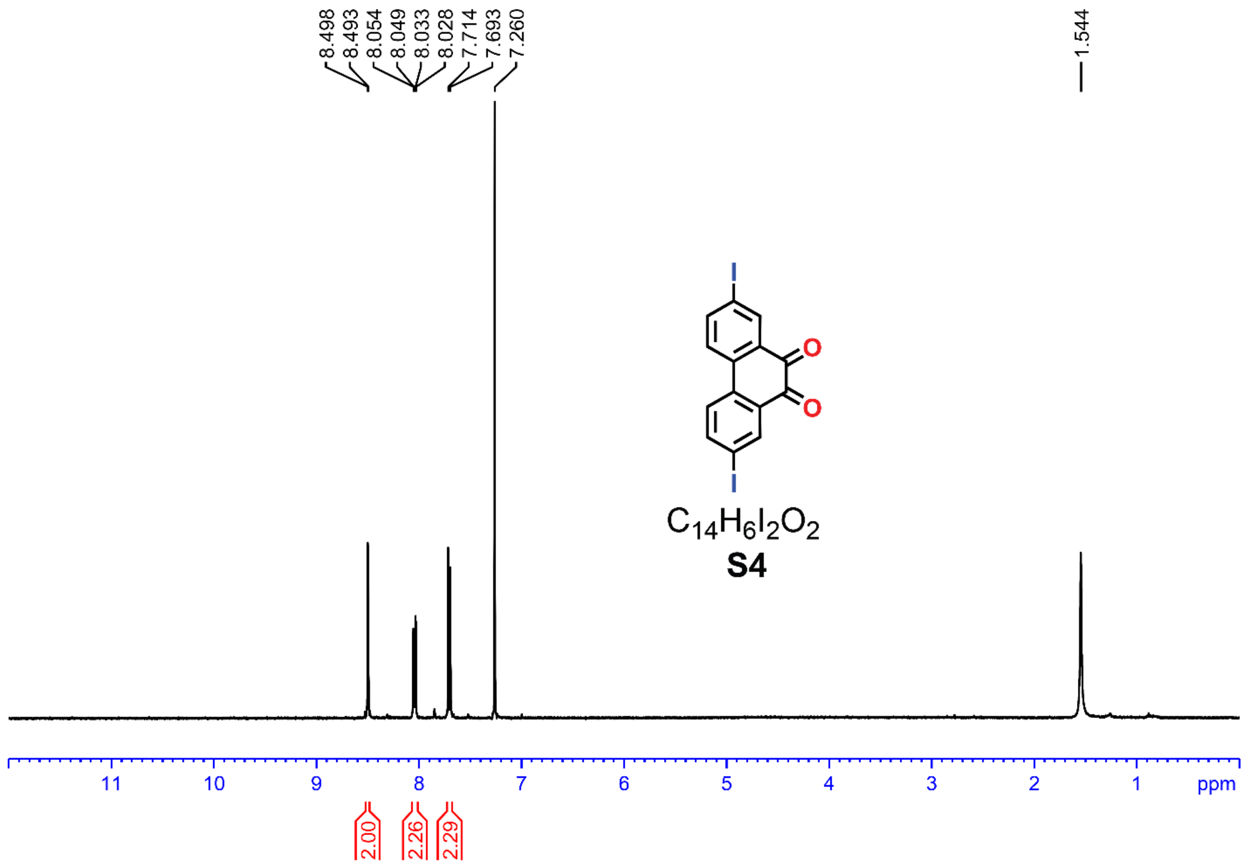

Supplementary Figure 3. ^{1}H NMR of compound **S4**.

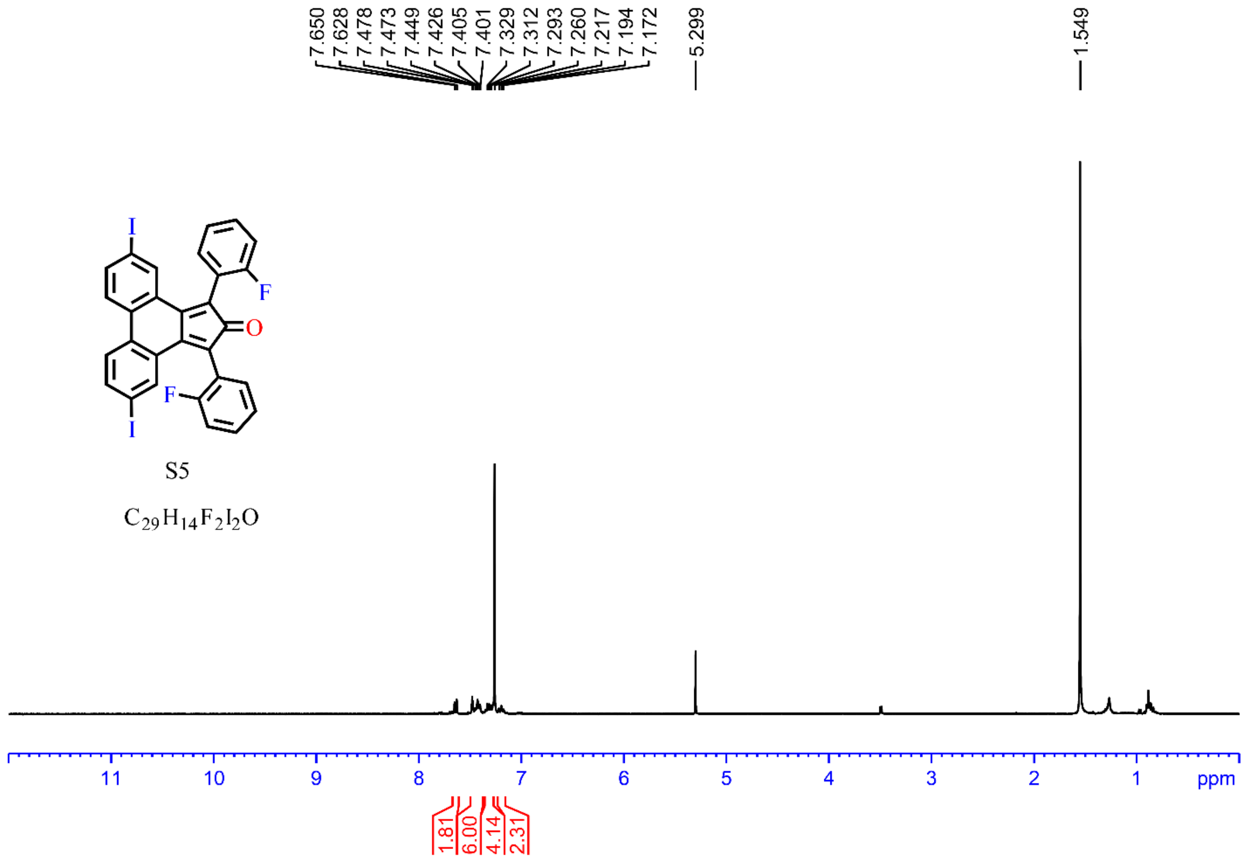

Supplementary Figure 4. ^{1}H NMR of compound **S5**.

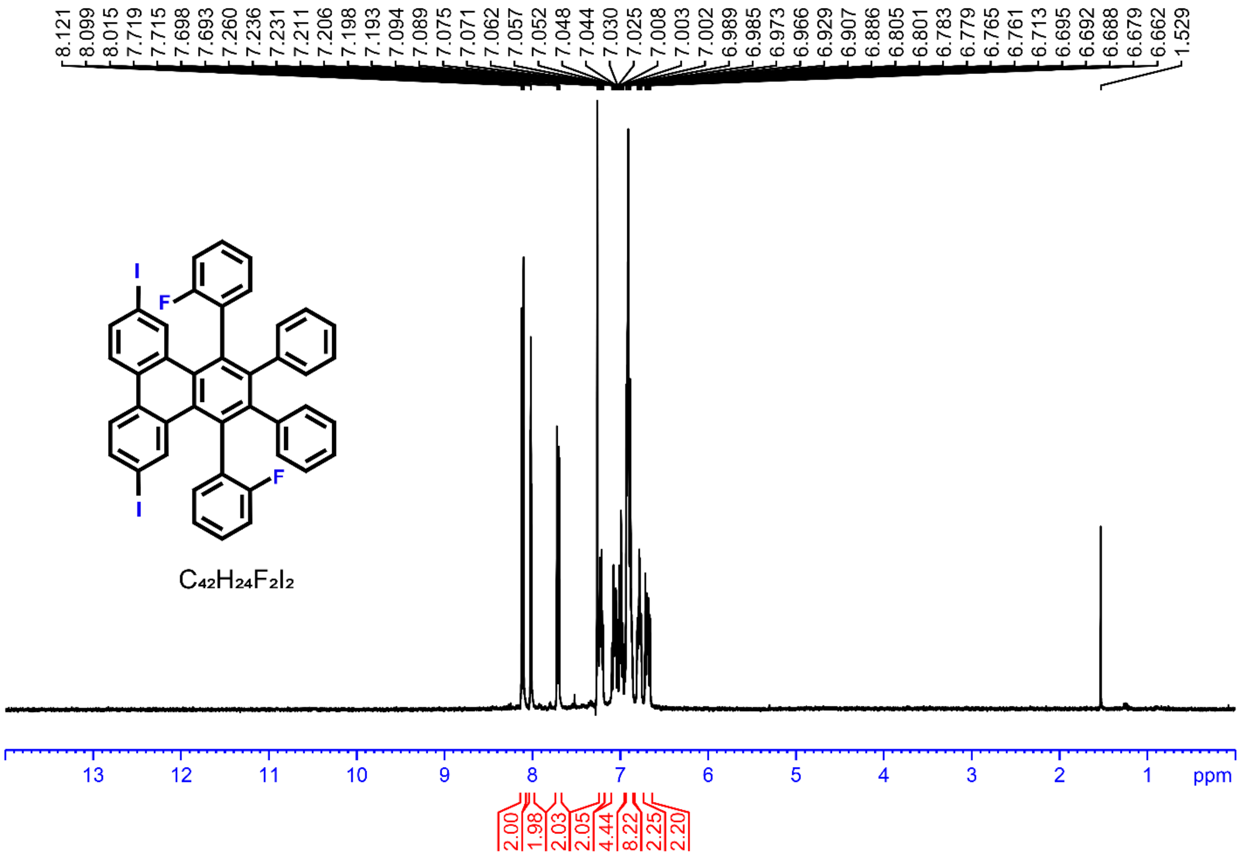

Supplementary Figure 5. ^{1}H NMR of precursor **1**.

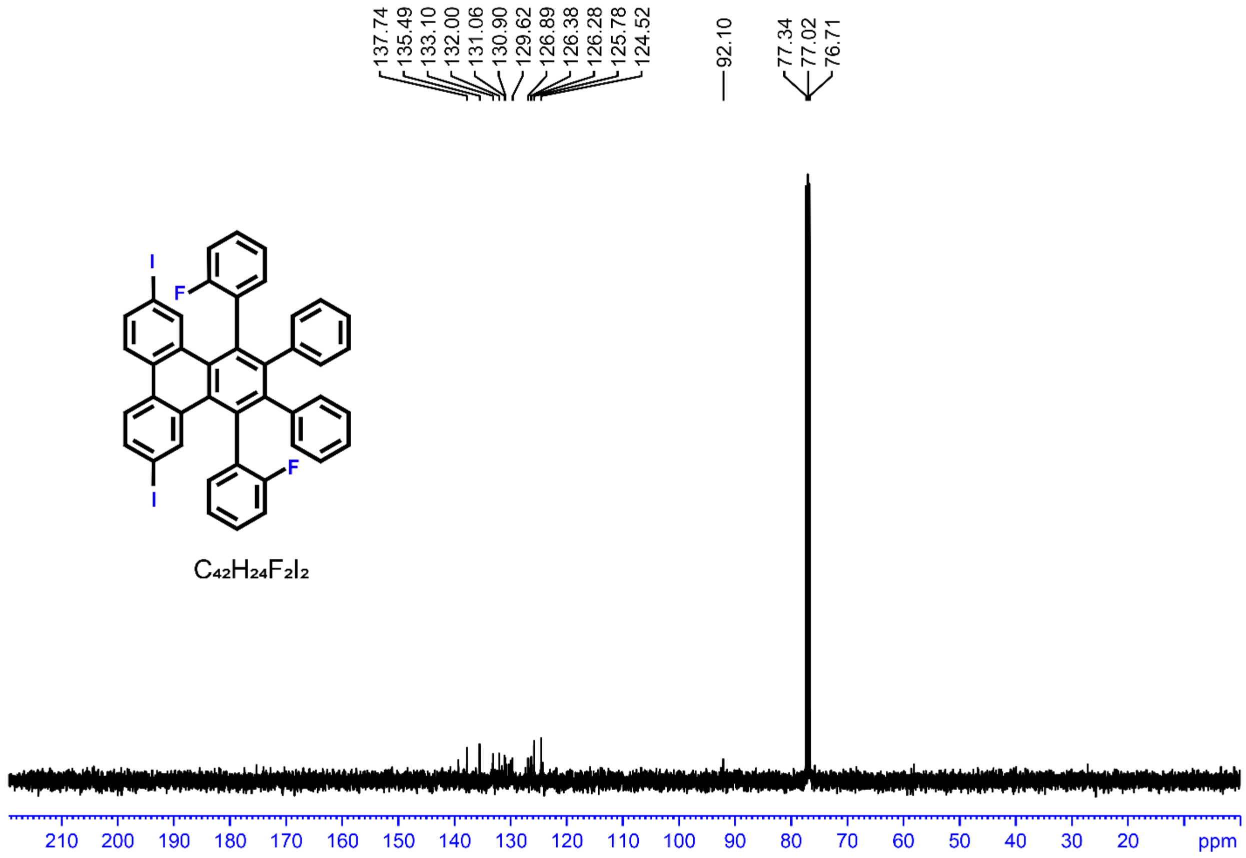

Supplementary Figure 6. ^{13}C NMR of precursor **1**.

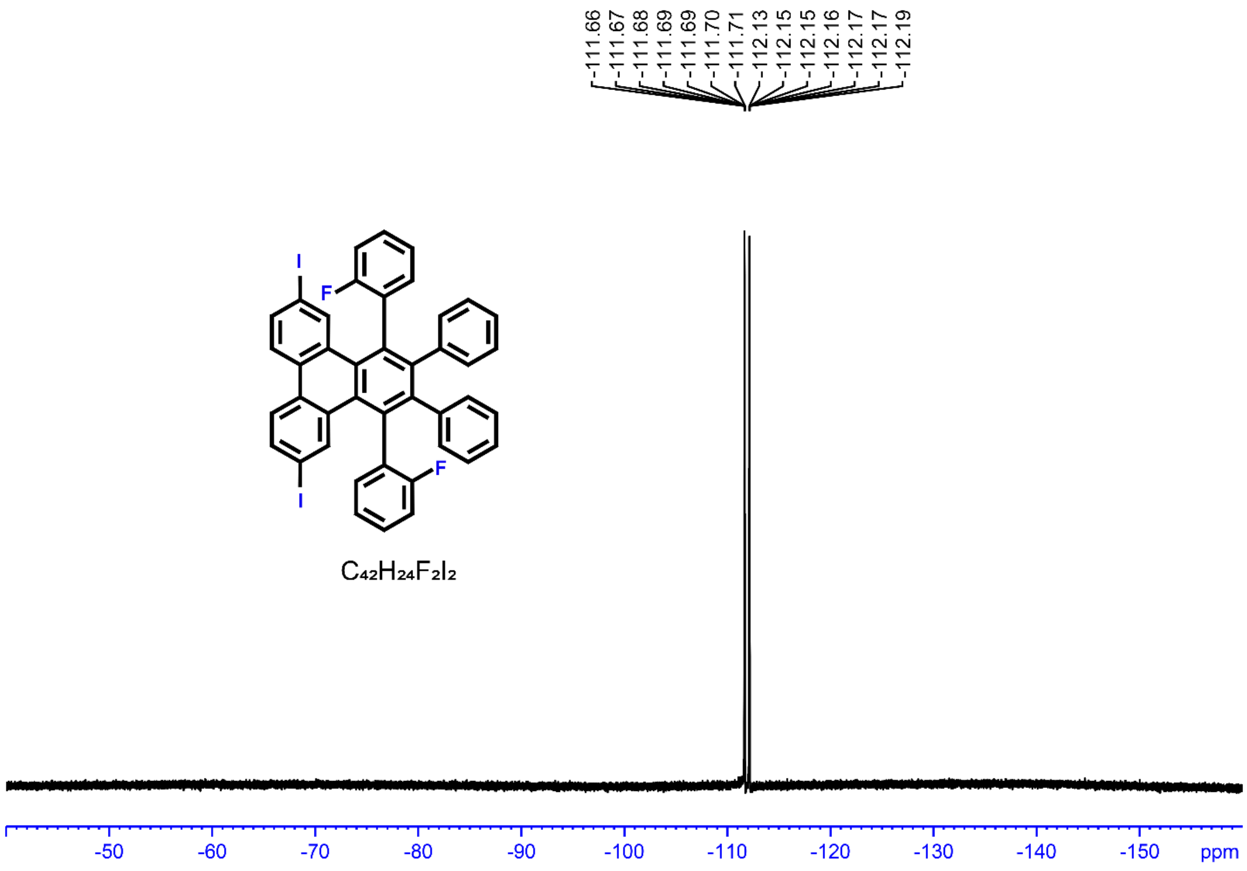

Supplementary Figure 7. ^{19}F NMR of precursor **1**.

3. Additional experimental and simulated scanning tunneling microscopy (STM) images

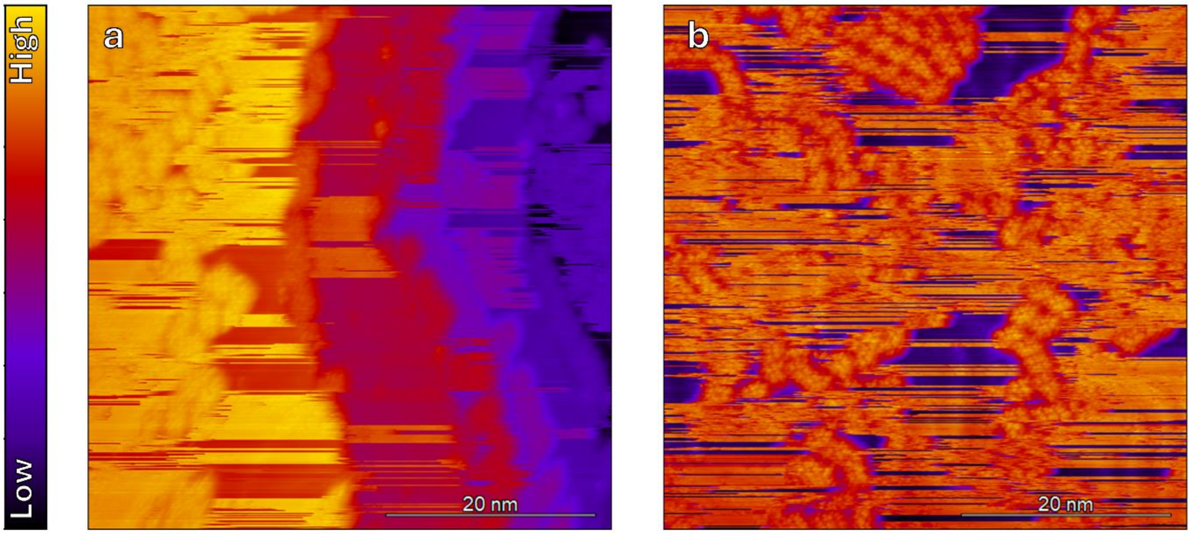

Supplementary Figure 8. Adsorption of pristine, non-fluorinated precursors to the surface occurs at RT on Au(111). In contrast to precursor **1**, the pristine chevron precursor adsorbs at RT on Au(111) along step edges (a) and on terraces (b). The large streaks are attributed to weak adsorption of the molecules on the surface, allowing the tip to push them around during scanning. Some clusters of precursors form on terraces. This cluster-like formation is previously observed for brominated and iodinated precursors on Au(111), and is likely dominated by $\pi - \pi$ interactions (see Refs. 40-43 in the main text). Scan parameters: -0.5V, 500pA.

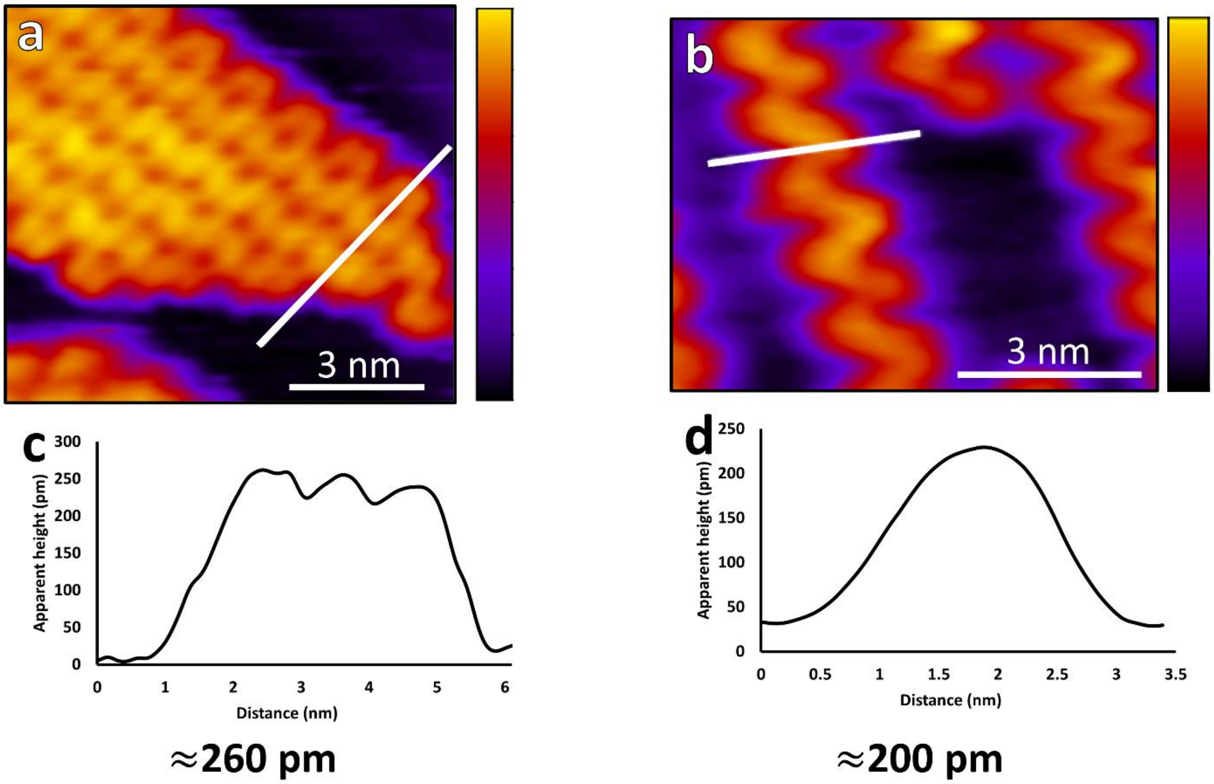

Supplementary Figure 9. Height profiles for polymers and GNRs grown from precursor **1.** STM images for polymers (a) and GNRs (b) grown from fluorinated precursor **1** on Au(111). Scans along the white lines give height profiles shown in (c) and (d), where the apparent heights are very close in value to the polymers arising from the pristine precursors on Au(111), which necessitated complementary investigative methods. Scan parameters: -1.5V, 50 pA for both (a) and (b).

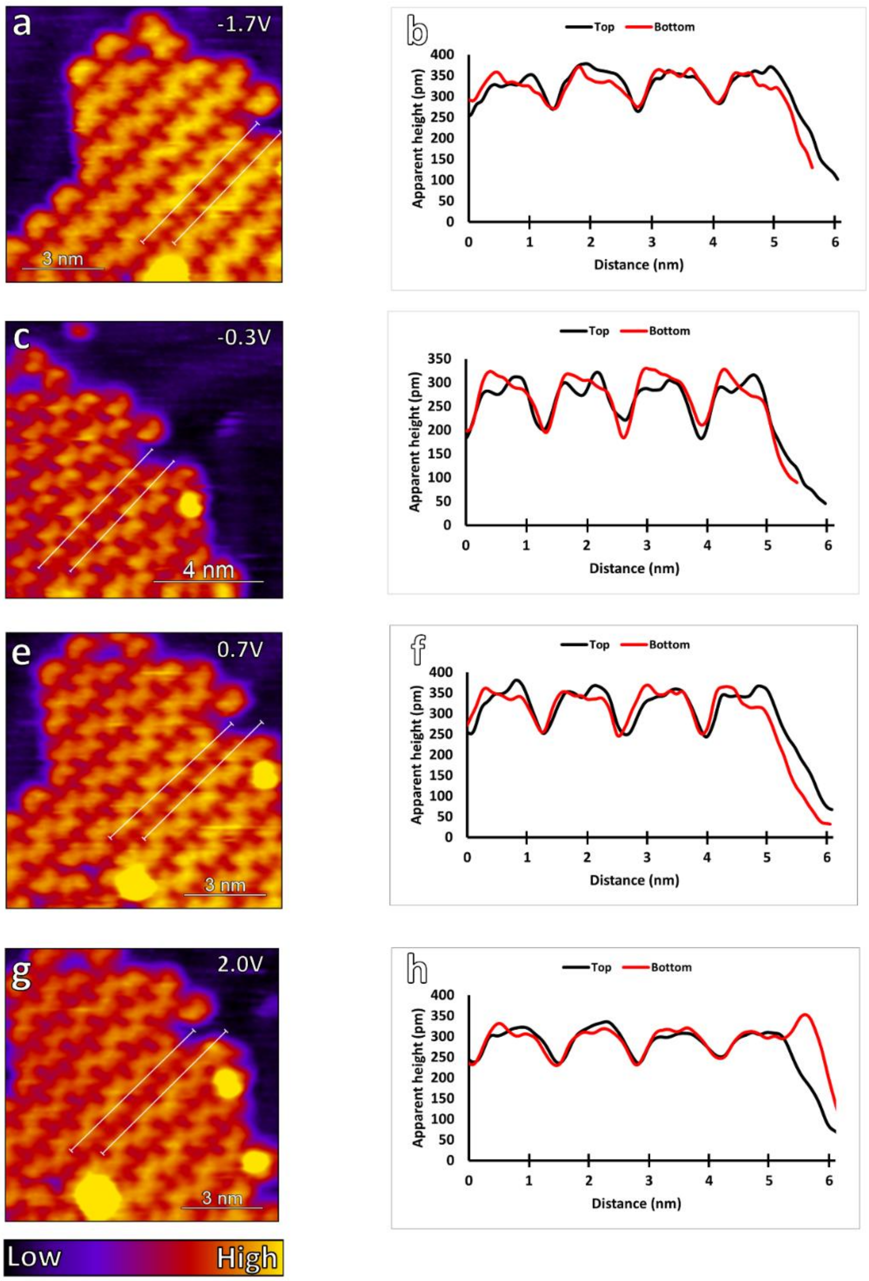

Supplementary Figure 10. Comparing line profiles across the top and bottom of the same polymer under various biases. All STM scans (a,c,e,g) acquired at 55pA current setpoint. The -0.3V scan (c) displays features most similar to the asymmetric peaks present in the simulated P-type polymer STM images seen in Supplementary Fig. 11a,b below, and so we assign it P-type helicity. As the magnitude of the bias increases, structural features are smeared out and the clarity of the asymmetric fringes decreases accordingly.

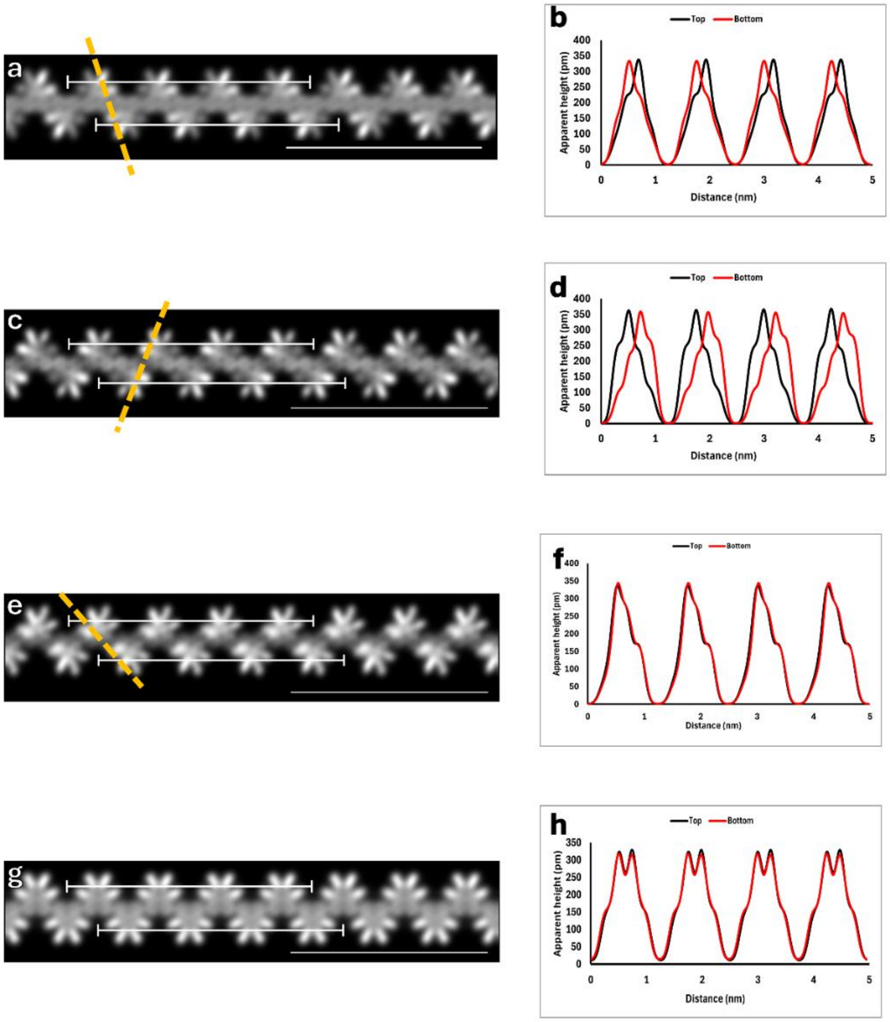

Supplementary Figure 11. Comparisons of polymers with different enforced helicities. (a,c,e,g) are simulated pristine chevron polymers with the following characteristics: (a) P-type; (c) M-type; (e) meso; (g) nonhelical. Yellow lines are added to highlight the asymmetrically taller parts of each subunit, and each scale bar is 4 nm. (b,d,f,h) are height profiles across the polymers in (a,c,e,g) and each type exhibits a characteristic profile. (b) P-type, as seen in the main text Fig. 4, has an asymmetric appearance with the top (bottom) phenyl fringe exhibiting a higher right (left) than left (right) apparent height. (d) M-type, not observed for our polymers, has an asymmetric appearance with the top (bottom) phenyl fringe exhibiting a higher left (right) than right (left) apparent height. (f) Meso-type, not observed for our polymers, has an asymmetric appearance with the top and

bottom phenyl fringes tilted such that the left side is greater in apparent height than the right. (h) Nonhelical chevron polymers exhibit symmetrical height profiles across the top and bottom phenyl fringes. Polymers scanned at higher absolute bias values tend to smear out and approach this in appearance (see Supplementary Fig. 10a,b,g,h).

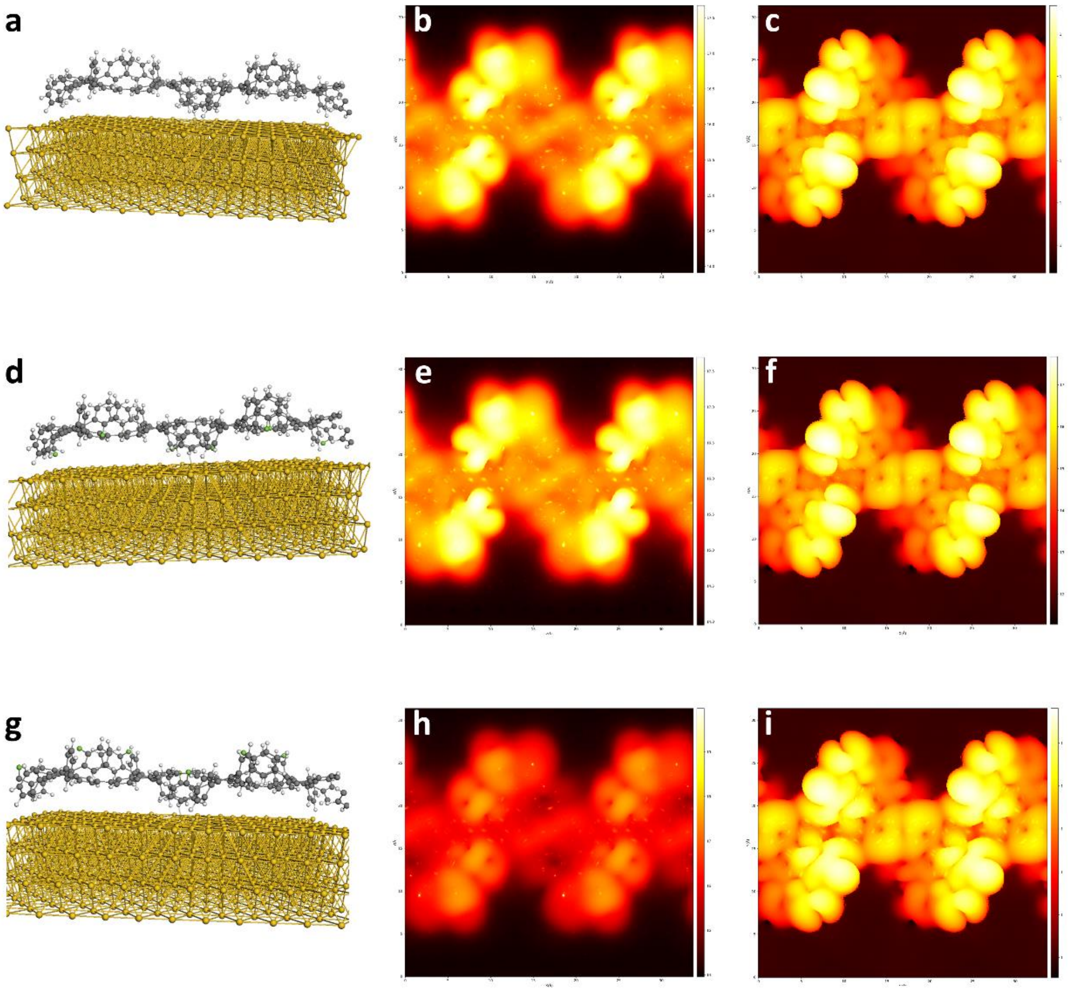

Supplementary Figure 12. Calculated adsorption geometries and simulated STM images for the pristine chevron precursor (a-c) and possible configurations of the fluorinated chevron precursor **1** (d-i). (d-f) represent a geometry with the fluorine atoms pointing towards the Au(111) surface, and (g-i) represent the fluorine atoms pointed away from the Au(111) surface. (b,e,h) are simulated at +1.0V, and (c,f,i) at -1.0V.

4. Additional XPS data

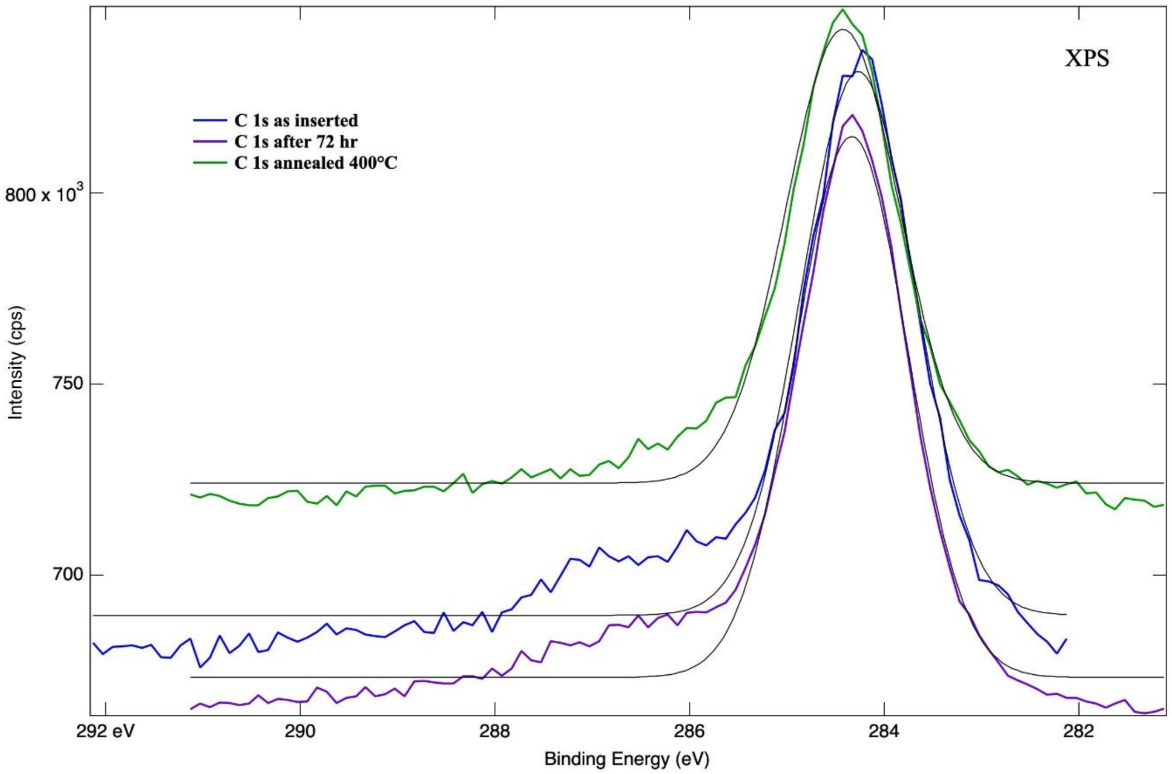

Supplementary Figure 13. Carbon 1s XPS spectra acquired under various conditions on a sample grown on an Au(111)/mica substrate. Annealing took place after 72hr in vacuum. Gray lines represent a Gaussian fit.

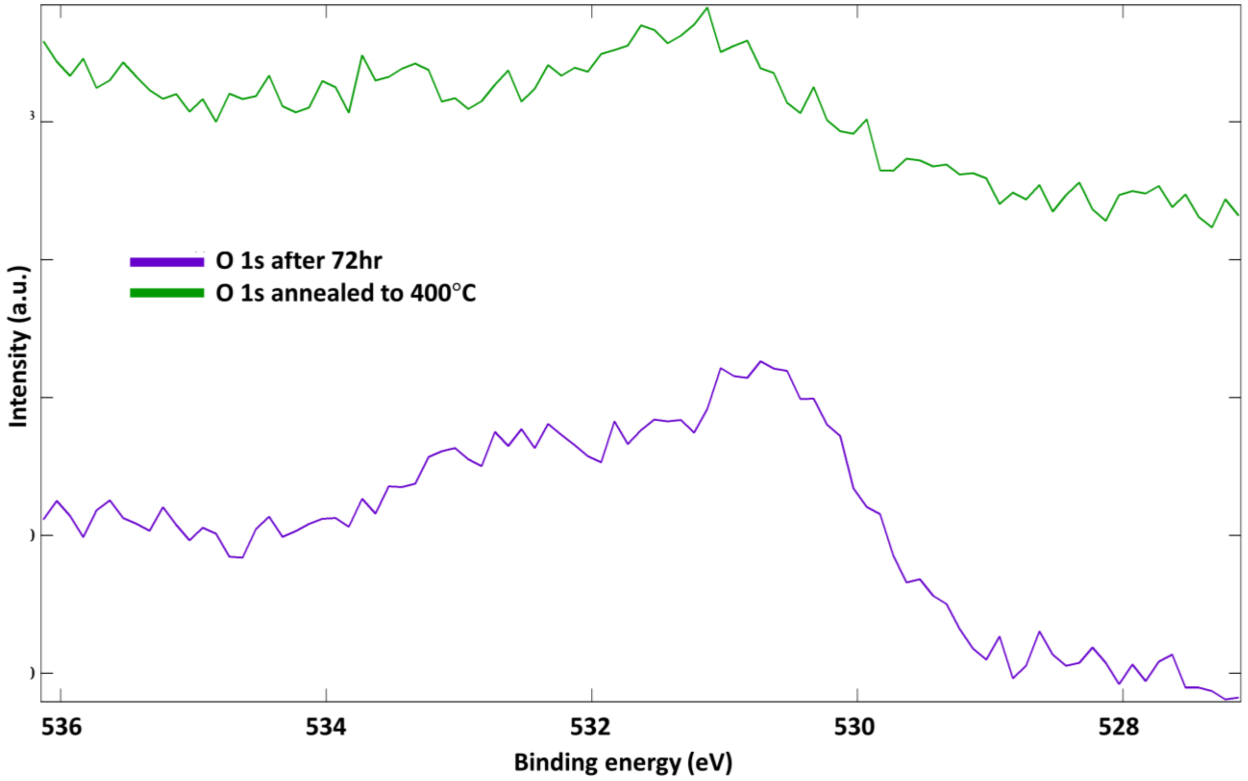

Supplementary Figure 14. Oxygen 1s XPS spectra acquired under various conditions on a sample grown on an Au(111)/mica substrate. Annealing took place after 72hr in vacuum.

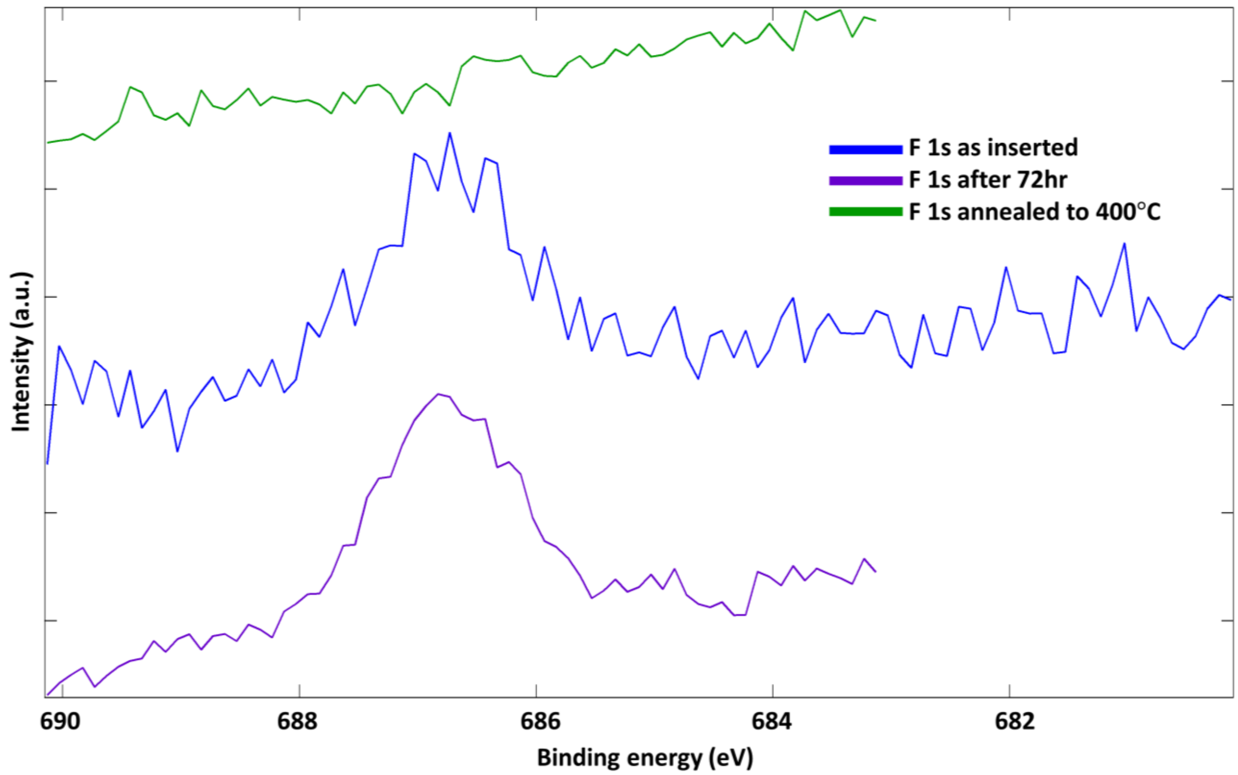

Supplementary Figure 15. Fluorine 1s XPS spectra acquired under various conditions on a sample grown on an Au(111)/mica substrate. Annealing took place after 72hr in vacuum.

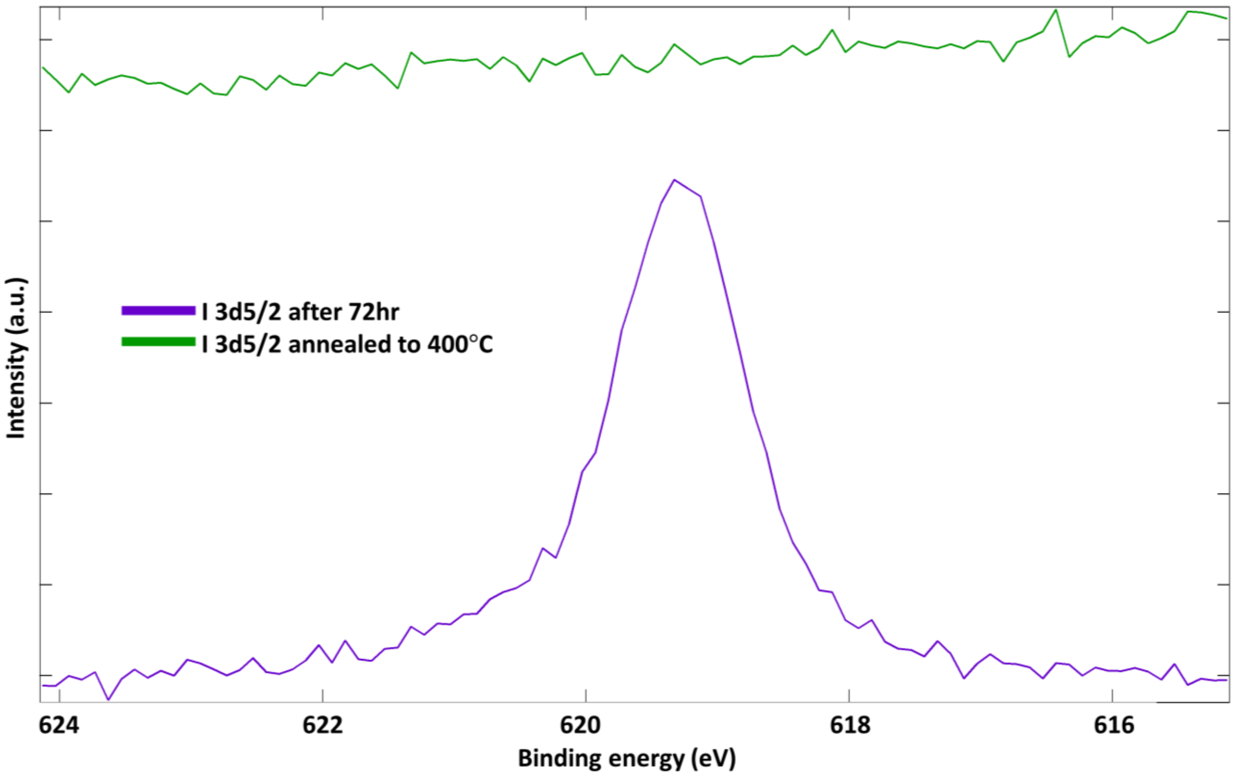

Supplementary Figure 16. Iodine 3d$^{5/2}$ XPS spectra acquired under various conditions on a sample grown on an Au(111)/mica substrate. Annealing took place after 72hr in vacuum.
