# Supplementary material for: Deposition temperature-mediated growth of helically shaped polymers and chevron-type graphene nanoribbons from a fluorinated precursor
Source: Commun Chem. 2024 Aug 31;7:193. doi: 10.1038/s42004-024-01253-9 (PMC11366011; doi:10.1038/s42004-024-01253-9)
Supplement: Supplementary file 3 — Description of Additional Supplementary Files [file 42004_2024_1253_MOESM3_ESM.pdf]

Description of Additional Supplementary Files

File name: Supplementary Data 1

Description: Optimized geometries of the polymer intermediates adsorbed on Au(111) surface. (a) Pristine polymer; (b) Fluorinated polymer with Fup; (c) Fluorinated polymer with Fdn.
