# Supplementary material for: Deposition temperature-mediated growth of helically shaped polymers and chevron-type graphene nanoribbons from a fluorinated precursor
Source: Commun Chem. 2024 Aug 31;7:193. doi: 10.1038/s42004-024-01253-9 (PMC11366011; doi:10.1038/s42004-024-01253-9)
Supplement: Supplementary file 4 — Supplementary Data 1 [file 42004_2024_1253_MOESM4_ESM.pdf]

Supplementary Data 1. Optimized geometries of the polymer intermediates adsorbed on Au(111) surface. (a) Pristine polymer; (b) Fluorinated polymer with F_{up}; (c) Fluorinated polymer with F_{dn}.

(a) Pristine polymer

424

Lattice=" 16.8784049 0.00000000 0.00000000 0.00000000 31.4190658 0.00000000 0.00000000 0.00000000 26.4960837"

C 0.73858469458 17.0062518837 13.0617760243
C 1.49800223490 18.1920272311 13.0678637275
C 2.84410621293 18.1642592387 12.8109320800
C 3.51875133157 16.9676904733 12.5031069453
C 2.80911766827 15.7442602499 12.5727547036
C 1.44271178222 15.8106704272 12.9044029189
C 4.91758696740 16.9698339068 12.1517094032
C 5.57440168958 18.1503410835 11.7535189971
C 6.92902823105 18.1840691493 11.5313909272
C 7.69717600545 17.0143872593 11.6638900969
C 7.02370120256 15.8219653310 11.9274094657
C 5.64927963311 15.7586884407 12.2141656461
C 4.94206942324 14.4931953684 12.4671022842
C 3.52862464122 14.4729550905 12.3490536137
C 5.60287184086 13.2631250402 12.7499749233
C 4.93988002909 12.0387044517 12.5941807704
C 3.59632394752 12.0107954243 12.1681163635
C 2.89205447017 13.2236692482 12.0679729511
C 6.96857595401 13.2383121297 13.3277951786
C 8.00404710106 12.5151203781 12.7158826313
C 9.23341620173 12.4833672618 13.3727563591
C 9.45171806911 13.1624325083 14.5850806758
C 8.42210332222 13.9029593103 15.1717607406
C 7.17806220146 13.9309376785 14.5414959294
C 5.61310493813 10.7391190420 12.8888149938
C 5.88194626323 10.3859305474 14.2132063028
C 6.42288365885 9.14283010120 14.5191022021
C 6.70795047004 8.23369972955 13.5035589037
C 6.44884551109 8.57729483375 12.1800946866
C 5.90172279647 9.82009649316 11.8759322239
C 2.92955401166 10.7116459118 11.8618936716
C 2.85962947104 9.65024724731 12.7759184434
C 2.19730574523 8.46350887665 12.4740038191
C 1.52088218189 8.32509959434 11.2712619602
C 1.54658188760 9.39081828452 10.3867336489
C 2.26603596283 10.5437883765 10.6467893176
C 1.52540023870 13.2586652768 11.5118856908
C 1.27469378611 14.2286381595 10.5182801681
C 16.8035005748 14.4751109179 10.2573228926

C	15.7672413261	13.6331467961	10.6957055411
C	16.0655174353	12.5394196250	11.5055579468
C	0.48985023940	12.4131878904	11.9788266076
C	15.3721404953	15.8410629275	13.0258354136
C	14.0297324093	15.8693653687	12.7510758998
C	15.4279785440	18.2248245893	12.9030944849
C	16.1311768074	17.0273162037	13.0510521854
C	14.0659790479	18.2916124725	12.5559176200
C	13.3483775138	19.5649609773	12.3503847310
C	11.9355207533	19.5490994332	12.4727017383
C	13.9894461182	20.8152070650	12.0893062380
C	13.2897228920	22.0191257656	12.2317018476
C	11.9538271945	21.9963595650	12.6643265793
C	11.2768658612	20.7771462684	12.7848773941
C	15.3456077190	20.8059506250	11.5119086632
C	15.6054903078	19.8321647774	10.5275376418
C	0.07962922494	19.6139905303	10.2466097714
C	1.10906856272	20.4602863364	10.6963441487
C	0.79902212472	21.5566236671	11.4947685199
C	16.3699979192	21.6746344437	11.9603152441
C	9.91674344719	20.8008792316	13.3763602227
C	9.71419829251	20.0454240220	14.5535725423
C	8.48034179592	20.0510594284	15.2024242848
C	7.44987718095	20.8278209394	14.6702314065
C	7.66167054466	21.5793402431	13.5005819745
C	8.88534447980	21.5819648313	12.8291645253
C	13.9365076505	23.3265510379	11.9323634801
C	14.2546298219	23.6307076271	10.6129961994
C	14.8808281893	24.8241111240	10.3037188188
C	15.2103929609	25.7553371781	11.2717055668
C	14.8736520041	25.4730098093	12.5931327207
C	14.2475666032	24.2732403247	12.9138147904
C	11.3195444979	23.3015147827	13.0100566393
C	11.0675953445	23.6160017303	14.3448144547
C	10.5209011330	24.8449932562	14.6912701734
C	10.2207415321	25.7824178431	13.7056359812
C	10.4846661011	25.4851143607	12.3704776029
C	11.0346380188	24.2549664498	12.0265929568
C	13.3573579558	17.0705890476	12.4564341080
C	11.9602372846	17.0791132061	12.1010199433
C	11.2284356729	18.2886344337	12.1969501321
C	11.3008042820	15.9087940974	11.6770179218
C	9.94330835499	15.8778143775	11.4773494578
C	9.85200486243	18.2315020825	11.9228318349
C	9.17442905517	17.0429718093	11.6485353513
H	1.03569390806	19.1480276827	13.2692666932
H	3.39654235014	19.0951882481	12.8430021444
H	0.90144940625	14.8898753706	13.0011133440

H	5.00722057738	19.0593905003	11.6025330938
H	7.38913461037	19.1205495563	11.2403046364
H	7.59874102922	14.9165860344	11.9427420452
H	7.84008526347	12.0287823766	11.7621671277
H	10.0576368039	11.9233870531	12.9391322038
H	10.4277959263	13.1310950471	15.0830817408
H	8.57878753532	14.4462998926	16.0993717674
H	6.36565747831	14.5096894109	14.9951147878
H	5.64586856290	11.0892756514	15.0043015267
H	6.61672980602	8.88522864649	15.5539030856
H	7.13127686673	7.26355378354	13.7410321955
H	6.66903582551	7.87577285829	11.3819651400
H	5.69174834420	10.0709482774	10.8410104343
H	3.32666603142	9.74665452235	13.7424489499
H	2.19645003652	7.65095113323	13.1915436755
H	0.97366384893	7.42378420264	11.0150441004
H	0.95817100740	9.33258172548	9.47951096849
H	2.24786128231	11.3596814609	9.93445569617
H	2.07612573362	14.7967812785	10.0628294330
H	16.5130361820	15.3958121348	9.75102713437
H	14.7267988705	13.8559052513	10.4489300136
H	15.2906463846	11.8360537974	11.8016268887
H	0.69376209742	11.6335714376	12.7135037192
H	15.8343754386	14.8826905528	13.2164962883
H	13.4778770392	14.9373796568	12.7606885593
H	15.9624484268	19.1476398130	13.0232223508
H	14.8133924137	19.2520314909	10.0726228864
H	0.36320589839	18.7059469056	9.72160560182
H	2.14893971637	20.2621687426	10.4246004861
H	1.56420630088	22.2714829476	11.7834827869
H	16.1611165312	22.4576189419	12.6878062339
H	10.5267727428	19.4376024277	14.9658933724
H	8.33445545093	19.4614700540	16.1040053120
H	6.47843833141	20.8314425748	15.1805612779
H	6.83716883978	22.1703391674	13.1107955185
H	9.04135698797	22.1489223108	11.9191870576
H	14.0176472194	22.9133443385	9.83364503386
H	15.1248094679	25.0467818915	9.27011343778
H	15.7053283382	26.6792147018	10.9933221587
H	15.1001126450	26.1961716554	13.3634754783
H	13.9972137805	24.0722735526	13.9463149394
H	11.3065250768	22.8899373541	15.1151584202
H	10.3302674266	25.0674087904	15.7338177571
H	9.78409229152	26.7374386894	13.9712025635
H	10.2569723846	26.2090189752	11.5951840457
H	11.2389239681	24.0356274587	10.9838186074
H	11.8684510275	15.0043964542	11.4970969810
H	9.48173951161	14.9479437371	11.1680042053

H	9.27900329507	19.1377519792	11.9633377394
Au	0.00000000000	0.00000000000	0.24315498000
Au	1.40653320000	2.61825445000	0.24315498000
Au	2.81306635000	0.00000000000	0.24315498000
Au	4.21959956000	2.61825445000	0.24315498000
Au	2.81306635000	5.23650889000	0.24315498000
Au	4.21959956000	7.85476334000	0.24315498000
Au	5.62613271000	5.23650889000	0.24315498000
Au	7.03266591000	7.85476334000	0.24315498000
Au	5.62613271000	10.4730177900	0.24315498000
Au	7.03266591000	13.0912722300	0.24315498000
Au	8.43919906000	10.4730177900	0.24315498000
Au	9.84573227000	13.0912722300	0.24315498000
Au	8.43919906000	15.7095266200	0.24315498000
Au	9.84573227000	18.3277810700	0.24315498000
Au	11.2522654200	15.7095266200	0.24315498000
Au	12.6587986200	18.3277810700	0.24315498000
Au	11.2522654200	20.9460355200	0.24315498000
Au	12.6587986200	23.5642899600	0.24315498000
Au	14.0653318300	20.9460355200	0.24315498000
Au	15.4718649800	23.5642899600	0.24315498000
Au	14.0653318300	26.1825444100	0.24315498000
Au	15.4718649800	28.8007988600	0.24315498000
Au	0.00000000000	26.1825444100	0.24315498000
Au	1.40653320000	28.8007988600	0.24315498000
Au	5.62613271000	0.00000000000	0.24315498000
Au	7.03266591000	2.61825445000	0.24315498000
Au	8.43919906000	0.00000000000	0.24315498000
Au	9.84573227000	2.61825445000	0.24315498000
Au	8.43919906000	5.23650889000	0.24315498000
Au	9.84573227000	7.85476334000	0.24315498000
Au	11.2522654200	5.23650889000	0.24315498000
Au	12.6587986200	7.85476334000	0.24315498000
Au	11.2522654200	10.4730177900	0.24315498000
Au	12.6587986200	13.0912722300	0.24315498000
Au	14.0653318300	10.4730177900	0.24315498000
Au	15.4718649800	13.0912722300	0.24315498000
Au	14.0653318300	15.7095266200	0.24315498000
Au	15.4718649800	18.3277810700	0.24315498000
Au	0.00000000000	15.7095266200	0.24315498000
Au	1.40653320000	18.3277810700	0.24315498000
Au	0.00000000000	20.9460355200	0.24315498000
Au	1.40653320000	23.5642899600	0.24315498000
Au	2.81306635000	20.9460355200	0.24315498000
Au	4.21959956000	23.5642899600	0.24315498000
Au	2.81306635000	26.1825444100	0.24315498000
Au	4.21959956000	28.8007988600	0.24315498000
Au	5.62613271000	26.1825444100	0.24315498000

Au	7.03266591000	28.8007988600	0.24315498000
Au	11.2522654200	0.00000000000	0.24315498000
Au	12.6587986200	2.61825445000	0.24315498000
Au	14.0653318300	0.00000000000	0.24315498000
Au	15.4718649800	2.61825445000	0.24315498000
Au	14.0653318300	5.23650889000	0.24315498000
Au	15.4718649800	7.85476334000	0.24315498000
Au	0.00000000000	5.23650889000	0.24315498000
Au	1.40653320000	7.85476334000	0.24315498000
Au	0.00000000000	10.4730177900	0.24315498000
Au	1.40653320000	13.0912722300	0.24315498000
Au	2.81306635000	10.4730177900	0.24315498000
Au	4.21959956000	13.0912722300	0.24315498000
Au	2.81306635000	15.7095266200	0.24315498000
Au	4.21959956000	18.3277810700	0.24315498000
Au	5.62613271000	15.7095266200	0.24315498000
Au	7.03266591000	18.3277810700	0.24315498000
Au	5.62613271000	20.9460355200	0.24315498000
Au	7.03266591000	23.5642899600	0.24315498000
Au	8.43919906000	20.9460355200	0.24315498000
Au	9.84573227000	23.5642899600	0.24315498000
Au	8.43919906000	26.1825444100	0.24315498000
Au	9.84573227000	28.8007988600	0.24315498000
Au	11.2522654200	26.1825444100	0.24315498000
Au	12.6587986200	28.8007988600	0.24315498000
Au	5.62613271000	3.49100591000	2.71166890000
Au	4.21959956000	0.87275146000	2.71166890000
Au	2.81306635000	3.49100591000	2.71166890000
Au	1.40653320000	0.87275146000	2.71166890000
Au	8.43919906000	8.72751480000	2.71166890000
Au	7.03266591000	6.10926036000	2.71166890000
Au	5.62613271000	8.72751480000	2.71166890000
Au	4.21959956000	6.10926036000	2.71166890000
Au	11.2522654200	13.9640237000	2.71166890000
Au	9.84573227000	11.3457692500	2.71166890000
Au	8.43919906000	13.9640237000	2.71166890000
Au	7.03266591000	11.3457692500	2.71166890000
Au	14.0653318300	19.2005325400	2.71166890000
Au	12.6587986200	16.5822780900	2.71166890000
Au	11.2522654200	19.2005325400	2.71166890000
Au	9.84573227000	16.5822780900	2.71166890000
Au	0.00000000000	24.4370414300	2.71166890000
Au	15.4718649800	21.8187869800	2.71166890000
Au	14.0653318300	24.4370414300	2.71166890000
Au	12.6587986200	21.8187869800	2.71166890000
Au	2.81306635000	29.6735503200	2.71166890000
Au	1.40653320000	27.0552958700	2.71166890000
Au	0.00000000000	29.6735503200	2.71166890000

Au	15.4718649800	27.0552958700	2.71166890000
Au	11.2522654200	3.49100591000	2.71166890000
Au	9.84573227000	0.87275146000	2.71166890000
Au	8.43919906000	3.49100591000	2.71166890000
Au	7.03266591000	0.87275146000	2.71166890000
Au	14.0653318300	8.72751480000	2.71166890000
Au	12.6587986200	6.10926036000	2.71166890000
Au	11.2522654200	8.72751480000	2.71166890000
Au	9.84573227000	6.10926036000	2.71166890000
Au	0.00000000000	13.9640237000	2.71166890000
Au	15.4718649800	11.3457692500	2.71166890000
Au	14.0653318300	13.9640237000	2.71166890000
Au	12.6587986200	11.3457692500	2.71166890000
Au	2.81306635000	19.2005325400	2.71166890000
Au	1.40653320000	16.5822780900	2.71166890000
Au	0.00000000000	19.2005325400	2.71166890000
Au	15.4718649800	16.5822780900	2.71166890000
Au	5.62613271000	24.4370414300	2.71166890000
Au	4.21959956000	21.8187869800	2.71166890000
Au	2.81306635000	24.4370414300	2.71166890000
Au	1.40653320000	21.8187869800	2.71166890000
Au	8.43919906000	29.6735503200	2.71166890000
Au	7.03266591000	27.0552958700	2.71166890000
Au	5.62613271000	29.6735503200	2.71166890000
Au	4.21959956000	27.0552958700	2.71166890000
Au	0.00000000000	3.49100591000	2.71166890000
Au	15.4718649800	0.87275146000	2.71166890000
Au	14.0653318300	3.49100591000	2.71166890000
Au	12.6587986200	0.87275146000	2.71166890000
Au	2.81306635000	8.72751480000	2.71166890000
Au	1.40653320000	6.10926036000	2.71166890000
Au	0.00000000000	8.72751480000	2.71166890000
Au	15.4718649800	6.10926036000	2.71166890000
Au	5.62613271000	13.9640237000	2.71166890000
Au	4.21959956000	11.3457692500	2.71166890000
Au	2.81306635000	13.9640237000	2.71166890000
Au	1.40653320000	11.3457692500	2.71166890000
Au	8.43919906000	19.2005325400	2.71166890000
Au	7.03266591000	16.5822780900	2.71166890000
Au	5.62613271000	19.2005325400	2.71166890000
Au	4.21959956000	16.5822780900	2.71166890000
Au	11.2522654200	24.4370414300	2.71166890000
Au	9.84573227000	21.8187869800	2.71166890000
Au	8.43919906000	24.4370414300	2.71166890000
Au	7.03266591000	21.8187869800	2.71166890000
Au	14.0653317700	29.6735503200	2.71166890000
Au	12.6587986200	27.0552958700	2.71166890000
Au	11.2522654200	29.6735503200	2.71166890000

Au	9.84573227000	27.0552958700	2.71166890000
Au	2.81306635000	1.74550293000	5.18018287000
Au	4.21959956000	4.36375743000	5.18018287000
Au	5.62613271000	1.74550293000	5.18018287000
Au	7.03266591000	4.36375743000	5.18018287000
Au	5.62613271000	6.98201182000	5.18018287000
Au	7.03266591000	9.60026627000	5.18018287000
Au	8.43919906000	6.98201182000	5.18018287000
Au	9.84573227000	9.60026627000	5.18018287000
Au	8.43919906000	12.2185207100	5.18018287000
Au	9.84573227000	14.8367751600	5.18018287000
Au	11.2522654200	12.2185207100	5.18018287000
Au	12.6587986200	14.8367751600	5.18018287000
Au	11.2522654200	17.4550296100	5.18018287000
Au	12.6587986200	20.0732840500	5.18018287000
Au	14.0653318300	17.4550296100	5.18018287000
Au	15.4718649800	20.0732840500	5.18018287000
Au	14.0653318300	22.6915385000	5.18018287000
Au	15.4718649800	25.3097928900	5.18018287000
Au	0.00000000000	22.6915385000	5.18018287000
Au	1.40653320000	25.3097928900	5.18018287000
Au	0.00000000000	27.9280473400	5.18018287000
Au	1.40653320000	30.5463017900	5.18018287000
Au	2.81306635000	27.9280473400	5.18018287000
Au	4.21959956000	30.5463017900	5.18018287000
Au	8.43919906000	1.74550293000	5.18018287000
Au	9.84573227000	4.36375743000	5.18018287000
Au	11.2522654200	1.74550293000	5.18018287000
Au	12.6587986200	4.36375743000	5.18018287000
Au	11.2522654200	6.98201182000	5.18018287000
Au	12.6587986200	9.60026627000	5.18018287000
Au	14.0653318300	6.98201182000	5.18018287000
Au	15.4718649800	9.60026627000	5.18018287000
Au	14.0653318300	12.2185207100	5.18018287000
Au	15.4718649800	14.8367751600	5.18018287000
Au	0.00000000000	12.2185207100	5.18018287000
Au	1.40653320000	14.8367751600	5.18018287000
Au	0.00000000000	17.4550296100	5.18018287000
Au	1.40653320000	20.0732840500	5.18018287000
Au	2.81306635000	17.4550296100	5.18018287000
Au	4.21959956000	20.0732840500	5.18018287000
Au	2.81306635000	22.6915385000	5.18018287000
Au	4.21959956000	25.3097928900	5.18018287000
Au	5.62613271000	22.6915385000	5.18018287000
Au	7.03266591000	25.3097928900	5.18018287000
Au	5.62613271000	27.9280473400	5.18018287000
Au	7.03266591000	30.5463017900	5.18018287000
Au	8.43919906000	27.9280473400	5.18018287000

Au	9.84573227000	30.5463017900	5.18018287000
Au	14.0653318300	1.74550293000	5.18018287000
Au	15.4718649800	4.36375743000	5.18018287000
Au	0.00000000000	1.74550293000	5.18018287000
Au	1.40653320000	4.36375743000	5.18018287000
Au	0.00000000000	6.98201182000	5.18018287000
Au	1.40653320000	9.60026627000	5.18018287000
Au	2.81306635000	6.98201182000	5.18018287000
Au	4.21959956000	9.60026627000	5.18018287000
Au	2.81306635000	12.2185207100	5.18018287000
Au	4.21959956000	14.8367751600	5.18018287000
Au	5.62613271000	12.2185207100	5.18018287000
Au	7.03266591000	14.8367751600	5.18018287000
Au	5.62613271000	17.4550296100	5.18018287000
Au	7.03266591000	20.0732840500	5.18018287000
Au	8.43919906000	17.4550296100	5.18018287000
Au	9.84573227000	20.0732840500	5.18018287000
Au	8.43919906000	22.6915385000	5.18018287000
Au	9.84573227000	25.3097928900	5.18018287000
Au	11.2522654200	22.6915385000	5.18018287000
Au	12.6587986200	25.3097928900	5.18018287000
Au	11.2522654200	27.9280473400	5.18018287000
Au	12.6587986200	30.5463017900	5.18018287000
Au	14.0653317700	27.9280473400	5.18018287000
Au	15.4718649800	30.5463017900	5.18018287000
Au	5.62970012289	31.4062628465	9.00570221028
Au	7.04315237167	2.57638452729	8.91791827472
Au	5.64385435095	5.12305003229	8.75685578622
Au	4.23473141600	2.54505885758	9.08833110573
Au	8.45446164943	5.18007033161	8.85883305524
Au	9.85337984017	7.76803101628	9.07420413581
Au	8.51878345387	10.3569072828	8.61671285065
Au	7.08306309303	7.73527991211	8.38787841857
Au	11.2726062966	10.3513211281	9.15989428137
Au	12.6377650361	12.8926077012	8.62593271265
Au	11.2943912875	15.5218484201	8.21528915515
Au	9.85299822169	12.9606611915	8.68222609604
Au	14.0647736460	15.5224320502	7.93778858701
Au	15.4268202621	18.2298377313	7.59018340436
Au	13.9887244204	21.0165211781	7.69316532940
Au	12.5820102756	18.2095313877	7.89073323544
Au	0.11607387514	21.0855731681	7.70339663165
Au	1.49261816721	23.8221547620	8.33599466441
Au	0.17004111763	26.5284187223	7.85568280086
Au	15.6214009209	23.6085671508	7.55894099309
Au	2.78248839638	26.3171167485	9.07262506258
Au	4.22189893974	28.8495519626	9.25525233425
Au	2.82503580464	0.00189252916	9.24643099901

Au	1.41021786669	28.8944469854	9.08513962045
Au	11.2428010408	0.05502136522	9.05061926084
Au	12.6560481030	2.61633066763	9.16951907288
Au	11.2545907732	5.19164784863	9.15254678998
Au	9.85386069321	2.61399911150	8.93234869445
Au	14.0687619811	5.15705971123	9.20355308505
Au	15.4459249099	7.62513925060	8.53740890253
Au	14.0489373355	10.2667868952	8.74615379807
Au	12.6832734198	7.74955770730	9.20980296916
Au	16.6914724773	10.2007654688	7.70682298611
Au	1.38995981326	12.8761991868	7.66354757701
Au	0.06881229952	15.7475802553	7.56504924116
Au	15.3308629224	12.7833028428	7.80110752034
Au	3.01451430934	15.9285228385	7.67223734698
Au	4.26543672669	18.5831803862	8.33464438980
Au	2.97941531932	21.2907816386	7.95878250615
Au	1.53724532871	18.4296998836	7.68475783948
Au	5.63861524941	21.1423366687	8.83005374911
Au	7.02519021552	23.6869267874	9.03213830968
Au	5.62316960247	26.2785624330	9.30816647821
Au	4.20576388463	23.7356923218	9.13316596388
Au	8.41748398744	26.2694845841	8.81227685899
Au	9.83625811885	28.8804199802	8.87628766207
Au	8.43542812456	0.01501866625	8.88295238968
Au	7.02439351860	28.8384616558	9.01594292065
Au	0.00415542048	0.02628791519	9.27597249351
Au	1.41652574386	2.55566788678	9.27032159584
Au	16.8756697710	5.08194655354	8.99094633635
Au	15.4683754823	2.58323376711	9.31974291661
Au	2.83026791040	5.05822437241	8.78931742607
Au	4.24970000071	7.60432446197	8.03573834285
Au	2.96253139410	10.3232935834	7.63617414442
Au	1.36082826045	7.34615690566	7.76509295895
Au	5.77774140942	10.3882117740	7.99827068393
Au	7.07302867166	13.0601097098	8.33576101552
Au	5.74725890738	15.7907340393	8.19546189112
Au	4.28107653325	13.1261640616	7.91537852283
Au	8.51943037327	15.6451258333	8.05693933922
Au	9.80879337751	18.3393480770	8.15154318608
Au	8.41984917361	21.0431048087	8.52675570516
Au	7.03083691291	18.4776779686	8.16891526588
Au	11.2039072825	20.9663330374	8.19163607257
Au	12.5171620413	23.7109291200	7.69802939190
Au	11.1931360567	26.3728665022	8.20142376972
Au	9.74234326029	23.6709291038	8.27707648485
Au	14.0101413882	26.6354152222	7.72609113950
Au	15.4864700665	28.9543920758	8.74391518036
Au	14.0630234698	0.05283249618	9.18840595528

Au 12.6473708392 28.9449278544 8.84490633564

(b) Fluorinated polymer with F_{up}

424
Lattice=" 16.8784049 0.00000000 0.00000000 0.00000000 31.4190658 0.00000000 0.00000000 0.00000000 26.4960837"
C 0.73920119150 16.9978487050 12.7061460360
C 1.49151141443 18.1761095460 12.8540643477
C 2.85206720428 18.1517654796 12.6503510572
C 3.51918715249 16.9733679517 12.2715599632
C 2.80826322994 15.7570536079 12.2106504205
C 1.42257221823 15.8138320899 12.4557497884
C 4.93017055241 16.9842708149 11.9812256274
C 5.58969990849 18.1709894838 11.6219454680
C 6.95124800768 18.2000854622 11.4185970189
C 7.70313280116 17.0226574031 11.5656055254
C 7.03621893768 15.8324784794 11.8232071798
C 5.65234839705 15.7742818746 12.0454211123
C 4.94403390878 14.5053341383 12.2241729996
C 3.54021152234 14.4926697208 12.0101672336
C 5.59764127567 13.2818584333 12.5409243051
C 4.92551141893 12.0688168364 12.3994902663
C 3.60715225381 12.0433492447 11.9086116019
C 2.91269861691 13.2457193359 11.7222768245
C 6.95424990588 13.2434732310 13.1528745789
C 8.02227086671 12.5091983119 12.6207678793
C 9.25641054206 12.4755626874 13.2644065461
C 9.45104013747 13.1788504364 14.4547621053
C 8.39727357002 13.9004767590 15.0162585094
C 7.18707517004 13.8976013537 14.3534075778
C 5.58103979631 10.7785621927 12.7499701149
C 5.81744941048 10.4622000053 14.0886713546
C 6.36213137725 9.22933879818 14.4352967192
C 6.67956145675 8.30126219860 13.4417758751
C 6.44517722437 8.61286507419 12.1012572737
C 5.89650669603 9.84584792623 11.7583141205
C 2.96303042469 10.7483786270 11.5885175092
C 2.87283148364 9.67642515085 12.4873962117
C 2.23731906248 8.48750588882 12.1358765746
C 1.61433371658 8.36316943213 10.8952393837
C 1.63606093324 9.45937324325 10.0483519333
C 2.33574556761 10.6153657046 10.3562051156
C 1.54541930270 13.2350981988 11.1547888953
C 1.22635474854 14.1635347428 10.1586626152
C 16.7726825859 14.4076346741 9.83580571520
C 15.7481277608 13.5577249911 10.2513602204
C 16.0658854368 12.5314885483 11.1363453498
C 0.47387633993 12.4899018402 11.6399191032

C	15.3832913319	15.8253681736	12.7971418721
C	14.0244999552	15.8564819893	12.5816622900
C	15.4547621576	18.2016495331	12.5034221173
C	16.1375112351	17.0083486445	12.7038528244
C	14.0738227720	18.2638575058	12.2368457604
C	13.3484415513	19.5323921259	12.0596085628
C	11.9471092077	19.5230151388	12.2693160879
C	13.9811816573	20.7784988561	11.7882002256
C	13.2951382089	21.9791940386	11.9841899870
C	11.9834785266	21.9579323657	12.4892231127
C	11.3051720509	20.7450596453	12.6152339431
C	15.3418345449	20.7899454071	11.2139138488
C	15.6767133066	19.8440058174	10.2504784510
C	0.12685694529	19.6542524742	9.87322669883
C	1.13248227948	20.5367766528	10.2873327091
C	0.80796986453	21.5486277376	11.1696731420
C	16.4007053067	21.5592331072	11.6778862616
C	9.95734268729	20.7708559841	13.2385911273
C	9.71726038851	20.0562439872	14.4043574601
C	8.51488024510	20.0600811351	15.0801827727
C	7.47208598124	20.8263007757	14.5615176665
C	7.66641742553	21.5654265733	13.3936531938
C	8.89685489714	21.5401119363	12.7438682344
C	13.9242697207	23.2848151091	11.6596961203
C	14.2551790571	23.5478573251	10.3402734315
C	14.8771988920	24.7469205139	10.0116479434
C	15.1751222710	25.7142057601	10.9526346429
C	14.8303147648	25.4603309729	12.2758173237
C	14.2141199634	24.2595792280	12.6192110652
C	11.3525851213	23.2507392637	12.8753267654
C	11.1193194972	23.5312549087	14.2227412153
C	10.5665477417	24.7516876957	14.6019957997
C	10.2396582307	25.7021943856	13.6344983027
C	10.4761998307	25.4291544214	12.2859012433
C	11.0343590183	24.2097987835	11.9095977093
C	13.3613365850	17.0484441638	12.2411022708
C	11.9520750301	17.0538750888	11.9433937812
C	11.2332217544	18.2628337867	12.0532501030
C	11.2891795672	15.8819973758	11.5430419943
C	9.92486328318	15.8619490652	11.3577562922
C	9.84817420182	18.2157745176	11.8382400377
C	9.17630158601	17.0343773893	11.5538300349
H	1.02082306475	19.1027737545	13.1388210662
H	3.42314423256	19.0526431597	12.8090222830
H	0.85409541282	14.9138069961	12.4317976611
H	5.01914300306	19.0750990481	11.4780694002
H	7.42294910406	19.1293772513	11.1412722881
H	7.61015031789	14.9342615691	11.8730396385

H	7.87627219268	11.9681734077	11.6985544169
H	10.0654550254	11.9008054845	12.8374423376
H	10.4103954192	13.1655837531	14.9487140980
H	8.50480055653	14.4457185051	15.9412014665
F	6.13942619362	14.5845505787	14.9235771974
H	5.55311634846	11.1747626761	14.8584234397
H	6.53383270204	8.99269294938	15.4744469173
H	7.10523462519	7.34709649940	13.7117194341
H	6.67731368913	7.89618214629	11.3263494717
H	5.69969572416	10.0777232550	10.7200242214
H	3.29090814469	9.77287002323	13.4728538330
H	2.19950186650	7.67419918230	12.8409228392
H	1.10666073467	7.46481280102	10.5775553191
H	1.05435700015	9.42849327907	9.14265311674
H	2.34488588264	11.4478200019	9.67042617710
H	1.99257346937	14.8453656626	9.82346669538
H	16.5038207245	15.3605824134	9.39048650083
H	14.7211990540	13.7866584640	10.0213658572
H	15.3048594593	11.8719379124	11.5214312546
F	0.65600383065	11.7521624809	12.7775395083
H	15.8507426141	14.8910750790	13.0594877178
H	13.4514209664	14.9511156541	12.7020832155
H	16.0189410070	19.1047566204	12.5339418947
H	14.9168034763	19.1598176528	9.90749146926
H	0.38990562409	18.7182902251	9.40481116084
H	2.15018900279	20.3831332738	9.96379409073
H	1.54281749072	22.2399513573	11.5514323643
F	16.2198256569	22.2979872352	12.8109701220
F	10.7359785290	19.2903827559	14.9225585987
H	8.41016803574	19.4754424528	15.9801917760
H	6.51877612427	20.8407766292	15.0679728174
H	6.86108999245	22.1599826034	12.9873167235
H	9.04561373343	22.1123605967	11.8402792660
H	14.0291928024	22.8081144331	9.58940787457
H	15.1612965641	24.9469590523	8.99303508286
H	15.6546054709	26.6352131878	10.6494258688
H	15.0515916983	26.1914638609	13.0337989597
H	13.9706502788	24.0690237856	13.6479655255
H	11.3749840661	22.7924533183	14.9696841218
H	10.3916078007	24.9561524074	15.6475348913
H	9.80493506558	26.6463204630	13.9262919416
H	10.2307925142	26.1623086804	11.5302970567
H	11.2293309864	24.0069482942	10.8661661789
H	11.8578103027	14.9834965381	11.3600041328
H	9.44717633696	14.9436486123	11.0563371873
H	9.27818097969	19.1143693339	11.9197938307
Au	0.00000000000	0.00000000000	0.24320000000
Au	1.40650000000	2.61830000000	0.24320000000

Au	2.81310000000	0.00000000000	0.24320000000
Au	4.21960000000	2.61830000000	0.24320000000
Au	2.81310000000	5.23650000000	0.24320000000
Au	4.21960000000	7.85480000000	0.24320000000
Au	5.62610000000	5.23650000000	0.24320000000
Au	7.03270000000	7.85480000000	0.24320000000
Au	5.62610000000	10.4730000000	0.24320000000
Au	7.03270000000	13.0913000000	0.24320000000
Au	8.43920000000	10.4730000000	0.24320000000
Au	9.84570000000	13.0913000000	0.24320000000
Au	8.43920000000	15.7095000000	0.24320000000
Au	9.84570000000	18.3278000000	0.24320000000
Au	11.2523000000	15.7095000000	0.24320000000
Au	12.6588000000	18.3278000000	0.24320000000
Au	11.2523000000	20.9460000000	0.24320000000
Au	12.6588000000	23.5643000000	0.24320000000
Au	14.0653000000	20.9460000000	0.24320000000
Au	15.4719000000	23.5643000000	0.24320000000
Au	14.0653000000	26.1826000000	0.24320000000
Au	15.4719000000	28.8008000000	0.24320000000
Au	0.00000000000	26.1826000000	0.24320000000
Au	1.40650000000	28.8008000000	0.24320000000
Au	5.62610000000	0.00000000000	0.24320000000
Au	7.03270000000	2.61830000000	0.24320000000
Au	8.43920000000	0.00000000000	0.24320000000
Au	9.84570000000	2.61830000000	0.24320000000
Au	8.43920000000	5.23650000000	0.24320000000
Au	9.84570000000	7.85480000000	0.24320000000
Au	11.2523000000	5.23650000000	0.24320000000
Au	12.6588000000	7.85480000000	0.24320000000
Au	11.2523000000	10.4730000000	0.24320000000
Au	12.6588000000	13.0913000000	0.24320000000
Au	14.0653000000	10.4730000000	0.24320000000
Au	15.4719000000	13.0913000000	0.24320000000
Au	14.0653000000	15.7095000000	0.24320000000
Au	15.4719000000	18.3278000000	0.24320000000
Au	0.00000000000	15.7095000000	0.24320000000
Au	1.40650000000	18.3278000000	0.24320000000
Au	0.00000000000	20.9460000000	0.24320000000
Au	1.40650000000	23.5643000000	0.24320000000
Au	2.81310000000	20.9460000000	0.24320000000
Au	4.21960000000	23.5643000000	0.24320000000
Au	2.81310000000	26.1826000000	0.24320000000
Au	4.21960000000	28.8008000000	0.24320000000
Au	5.62610000000	26.1826000000	0.24320000000
Au	7.03270000000	28.8008000000	0.24320000000
Au	11.2523000000	0.00000000000	0.24320000000
Au	12.6588000000	2.61830000000	0.24320000000

Au	14.0653000000	0.00000000000	0.24320000000
Au	15.4719000000	2.61830000000	0.24320000000
Au	14.0653000000	5.23650000000	0.24320000000
Au	15.4719000000	7.85480000000	0.24320000000
Au	0.00000000000	5.23650000000	0.24320000000
Au	1.40650000000	7.85480000000	0.24320000000
Au	0.00000000000	10.4730000000	0.24320000000
Au	1.40650000000	13.0913000000	0.24320000000
Au	2.81310000000	10.4730000000	0.24320000000
Au	4.21960000000	13.0913000000	0.24320000000
Au	2.81310000000	15.7095000000	0.24320000000
Au	4.21960000000	18.3278000000	0.24320000000
Au	5.62610000000	15.7095000000	0.24320000000
Au	7.03270000000	18.3278000000	0.24320000000
Au	5.62610000000	20.9460000000	0.24320000000
Au	7.03270000000	23.5643000000	0.24320000000
Au	8.43920000000	20.9460000000	0.24320000000
Au	9.84570000000	23.5643000000	0.24320000000
Au	8.43920000000	26.1826000000	0.24320000000
Au	9.84570000000	28.8008000000	0.24320000000
Au	11.2523000000	26.1826000000	0.24320000000
Au	12.6588000000	28.8008000000	0.24320000000
Au	5.62610000000	3.49100000000	2.71170000000
Au	4.21960000000	0.87280000000	2.71170000000
Au	2.81310000000	3.49100000000	2.71170000000
Au	1.40650000000	0.87280000000	2.71170000000
Au	8.43920000000	8.72750000000	2.71170000000
Au	7.03270000000	6.10930000000	2.71170000000
Au	5.62610000000	8.72750000000	2.71170000000
Au	4.21960000000	6.10930000000	2.71170000000
Au	11.2523000000	13.9640000000	2.71170000000
Au	9.84570000000	11.3458000000	2.71170000000
Au	8.43920000000	13.9640000000	2.71170000000
Au	7.03270000000	11.3458000000	2.71170000000
Au	14.0653000000	19.2005000000	2.71170000000
Au	12.6588000000	16.5823000000	2.71170000000
Au	11.2523000000	19.2005000000	2.71170000000
Au	9.84570000000	16.5823000000	2.71170000000
Au	0.00000000000	24.4371000000	2.71170000000
Au	15.4719000000	21.8188000000	2.71170000000
Au	14.0653000000	24.4371000000	2.71170000000
Au	12.6588000000	21.8188000000	2.71170000000
Au	2.81310000000	29.6736000000	2.71170000000
Au	1.40650000000	27.0553000000	2.71170000000
Au	0.00000000000	29.6736000000	2.71170000000
Au	15.4719000000	27.0553000000	2.71170000000
Au	11.2523000000	3.49100000000	2.71170000000
Au	9.84570000000	0.87280000000	2.71170000000

Au	8.43920000000	3.49100000000	2.71170000000
Au	7.03270000000	0.87280000000	2.71170000000
Au	14.0653000000	8.72750000000	2.71170000000
Au	12.6588000000	6.10930000000	2.71170000000
Au	11.2523000000	8.72750000000	2.71170000000
Au	9.84570000000	6.10930000000	2.71170000000
Au	0.00000000000	13.9640000000	2.71170000000
Au	15.4719000000	11.3458000000	2.71170000000
Au	14.0653000000	13.9640000000	2.71170000000
Au	12.6588000000	11.3458000000	2.71170000000
Au	2.81310000000	19.2005000000	2.71170000000
Au	1.40650000000	16.5823000000	2.71170000000
Au	0.00000000000	19.2005000000	2.71170000000
Au	15.4719000000	16.5823000000	2.71170000000
Au	5.62610000000	24.4371000000	2.71170000000
Au	4.21960000000	21.8188000000	2.71170000000
Au	2.81310000000	24.4371000000	2.71170000000
Au	1.40650000000	21.8188000000	2.71170000000
Au	8.43920000000	29.6736000000	2.71170000000
Au	7.03270000000	27.0553000000	2.71170000000
Au	5.62610000000	29.6736000000	2.71170000000
Au	4.21960000000	27.0553000000	2.71170000000
Au	0.00000000000	3.49100000000	2.71170000000
Au	15.4719000000	0.87280000000	2.71170000000
Au	14.0653000000	3.49100000000	2.71170000000
Au	12.6588000000	0.87280000000	2.71170000000
Au	2.81310000000	8.72750000000	2.71170000000
Au	1.40650000000	6.10930000000	2.71170000000
Au	0.00000000000	8.72750000000	2.71170000000
Au	15.4719000000	6.10930000000	2.71170000000
Au	5.62610000000	13.9640000000	2.71170000000
Au	4.21960000000	11.3458000000	2.71170000000
Au	2.81310000000	13.9640000000	2.71170000000
Au	1.40650000000	11.3458000000	2.71170000000
Au	8.43920000000	19.2005000000	2.71170000000
Au	7.03270000000	16.5823000000	2.71170000000
Au	5.62610000000	19.2005000000	2.71170000000
Au	4.21960000000	16.5823000000	2.71170000000
Au	11.2523000000	24.4371000000	2.71170000000
Au	9.84570000000	21.8188000000	2.71170000000
Au	8.43920000000	24.4371000000	2.71170000000
Au	7.03270000000	21.8188000000	2.71170000000
Au	14.0653000000	29.6736000000	2.71170000000
Au	12.6588000000	27.0553000000	2.71170000000
Au	11.2523000000	29.6736000000	2.71170000000
Au	9.84570000000	27.0553000000	2.71170000000
Au	2.81310000000	1.74550000000	5.18020000000
Au	4.21960000000	4.36380000000	5.18020000000

Au	5.62610000000	1.74550000000	5.18020000000
Au	7.03270000000	4.36380000000	5.18020000000
Au	5.62610000000	6.98200000000	5.18020000000
Au	7.03270000000	9.60030000000	5.18020000000
Au	8.43920000000	6.98200000000	5.18020000000
Au	9.84570000000	9.60030000000	5.18020000000
Au	8.43920000000	12.2185000000	5.18020000000
Au	9.84570000000	14.8368000000	5.18020000000
Au	11.2523000000	12.2185000000	5.18020000000
Au	12.6588000000	14.8368000000	5.18020000000
Au	11.2523000000	17.4550000000	5.18020000000
Au	12.6588000000	20.0733000000	5.18020000000
Au	14.0653000000	17.4550000000	5.18020000000
Au	15.4719000000	20.0733000000	5.18020000000
Au	14.0653000000	22.6915000000	5.18020000000
Au	15.4719000000	25.3098000000	5.18020000000
Au	0.00000000000	22.6915000000	5.18020000000
Au	1.40650000000	25.3098000000	5.18020000000
Au	0.00000000000	27.9281000000	5.18020000000
Au	1.40650000000	30.5463000000	5.18020000000
Au	2.81310000000	27.9281000000	5.18020000000
Au	4.21960000000	30.5463000000	5.18020000000
Au	8.43920000000	1.74550000000	5.18020000000
Au	9.84570000000	4.36380000000	5.18020000000
Au	11.2523000000	1.74550000000	5.18020000000
Au	12.6588000000	4.36380000000	5.18020000000
Au	11.2523000000	6.98200000000	5.18020000000
Au	12.6588000000	9.60030000000	5.18020000000
Au	14.0653000000	6.98200000000	5.18020000000
Au	15.4719000000	9.60030000000	5.18020000000
Au	14.0653000000	12.2185000000	5.18020000000
Au	15.4719000000	14.8368000000	5.18020000000
Au	0.00000000000	12.2185000000	5.18020000000
Au	1.40650000000	14.8368000000	5.18020000000
Au	0.00000000000	17.4550000000	5.18020000000
Au	1.40650000000	20.0733000000	5.18020000000
Au	2.81310000000	17.4550000000	5.18020000000
Au	4.21960000000	20.0733000000	5.18020000000
Au	2.81310000000	22.6915000000	5.18020000000
Au	4.21960000000	25.3098000000	5.18020000000
Au	5.62610000000	22.6915000000	5.18020000000
Au	7.03270000000	25.3098000000	5.18020000000
Au	5.62610000000	27.9281000000	5.18020000000
Au	7.03270000000	30.5463000000	5.18020000000
Au	8.43920000000	27.9281000000	5.18020000000
Au	9.84570000000	30.5463000000	5.18020000000
Au	14.0653000000	1.74550000000	5.18020000000
Au	15.4719000000	4.36380000000	5.18020000000

Au	0.00000000000	1.74550000000	5.18020000000
Au	1.40650000000	4.36380000000	5.18020000000
Au	0.00000000000	6.98200000000	5.18020000000
Au	1.40650000000	9.60030000000	5.18020000000
Au	2.81310000000	6.98200000000	5.18020000000
Au	4.21960000000	9.60030000000	5.18020000000
Au	2.81310000000	12.2185000000	5.18020000000
Au	4.21960000000	14.8368000000	5.18020000000
Au	5.62610000000	12.2185000000	5.18020000000
Au	7.03270000000	14.8368000000	5.18020000000
Au	5.62610000000	17.4550000000	5.18020000000
Au	7.03270000000	20.0733000000	5.18020000000
Au	8.43920000000	17.4550000000	5.18020000000
Au	9.84570000000	20.0733000000	5.18020000000
Au	8.43920000000	22.6915000000	5.18020000000
Au	9.84570000000	25.3098000000	5.18020000000
Au	11.2523000000	22.6915000000	5.18020000000
Au	12.6588000000	25.3098000000	5.18020000000
Au	11.2523000000	27.9281000000	5.18020000000
Au	12.6588000000	30.5463000000	5.18020000000
Au	14.0653000000	27.9281000000	5.18020000000
Au	15.4719000000	30.5463000000	5.18020000000
Au	5.62794102981	0.01161576839	7.73356207639
Au	7.03451231350	2.62918709427	7.72134729203
Au	5.63413191548	5.22764970181	7.74377083303
Au	4.22521641951	2.60488319995	7.73275685613
Au	8.44208908236	5.24854377260	7.73559111772
Au	9.85019546128	7.85806118164	7.72829651268
Au	8.46462993403	10.4790477694	7.75000476142
Au	7.04451426687	7.85326188643	7.73748429771
Au	11.2440998452	10.4714682474	7.74580712603
Au	12.6325716729	13.0878356177	7.71065921827
Au	11.2376829594	15.7129374786	7.75396281114
Au	9.84522566430	13.0949147978	7.73792481333
Au	14.0227955613	15.7279013706	7.64973025621
Au	15.3957021923	18.3211906907	7.52539652499
Au	14.0172318951	20.9510454652	7.62308088386
Au	12.6094757379	18.3355763340	7.75081043711
Au	0.02795725832	20.9924751333	7.61691325910
Au	1.45569179423	23.6447587549	7.71414349636
Au	0.04044108794	26.2874876108	7.62434846883
Au	15.5451124977	23.5054457098	7.45812572390
Au	2.83666301464	26.2362705899	7.73843239626
Au	4.22584599186	28.8259426942	7.72556566477
Au	2.81765332341	0.01679227178	7.73399197700
Au	1.41772424959	28.8588543660	7.73867547433
Au	11.2472139282	0.03483402572	7.72510554631
Au	12.6537996150	2.63559284235	7.72745345831

Au	11.2508447181	5.24703935694	7.73183381336
Au	9.84442334705	2.63801722984	7.72417424979
Au	14.0604137847	5.23096679575	7.73421824132
Au	15.4752756395	7.81586464992	7.71725012698
Au	14.0192699202	10.4311607280	7.75514260376
Au	12.6593311046	7.84232682225	7.73497339359
Au	16.8082060207	10.4723977463	7.54789448674
Au	1.44092025178	13.0754380016	7.58574832427
Au	0.02627492082	15.8515044337	7.45865740316
Au	15.4359342578	13.0255843818	7.59038251207
Au	2.89361813091	15.7762904372	7.63699454495
Au	4.28525884249	18.3764981819	7.74839610569
Au	2.88511312266	21.0292813351	7.61721063436
Au	1.50979043119	18.3440814569	7.53288552604
Au	5.66379442388	20.9777874907	7.74731346879
Au	7.03889145870	23.5807957744	7.73716350461
Au	5.63160177093	26.2066558956	7.72645306747
Au	4.24896535744	23.6123010568	7.74782230306
Au	8.42401800443	26.2056985258	7.73786658703
Au	9.83233247481	28.8279231100	7.72876073864
Au	8.43726298555	0.01770189276	7.72486356181
Au	7.02990244059	28.8212515123	7.72784303017
Au	0.00129387711	0.02796747589	7.73689760870
Au	1.40516280484	2.60025396194	7.73678024201
Au	16.8671292777	5.17503123726	7.75973573037
Au	15.4671181004	2.61700721754	7.73635250541
Au	2.82682984623	5.16444782885	7.75493934592
Au	4.24274253808	7.81518824710	7.69603047066
Au	2.91320895227	10.4748717563	7.51750610744
Au	1.42244384227	7.68789547035	7.56947715057
Au	5.69072162572	10.4844539991	7.72564638945
Au	7.05807700412	13.1019580956	7.74285713460
Au	5.67206309263	15.7379987972	7.75014424575
Au	4.26637941729	13.1357775043	7.69389957176
Au	8.45295508742	15.7172783148	7.72447591027
Au	9.83825384142	18.3385272151	7.73776566323
Au	8.44405861716	20.9638275052	7.74641722373
Au	7.06062709893	18.3460096988	7.73338276324
Au	11.2283097036	20.9571051856	7.74349932372
Au	12.6121215059	23.6053228856	7.58954863621
Au	11.2140391209	26.2339195747	7.71749706549
Au	9.82399247979	23.5848539453	7.73214919735
Au	14.0063450745	26.3411938771	7.56932108026
Au	15.4756875110	28.8735781244	7.73773726934
Au	14.0644649195	0.03871569645	7.73028017679
Au	12.6385011456	28.8841627236	7.74313850279

(c) Fluorinated polymer with F_{dn}

424
Lattice=" 16.8784049 0.00000000 0.00000000 0.00000000 31.4190658 0.00000000 0.00000000 0.00000000 26.4960837"
C 0.739121609083 17.0074925796 13.0565045287
C 1.49790193596 18.1864252401 13.0666983400
C 2.84589664668 18.1591150881 12.8097283136
C 3.51922319175 16.9684540569 12.5014108962
C 2.81267057499 15.7462340178 12.5729511337
C 1.44568753355 15.8153171146 12.8989913828
C 4.91809055378 16.9705973342 12.1505076211
C 5.57405242708 18.1458007618 11.7540739889
C 6.92977064630 18.1787897401 11.5322427169
C 7.69712794089 17.0153732481 11.6643708889
C 7.02044601467 15.8295855561 11.9273820538
C 5.64650920113 15.7615597989 12.2128126893
C 4.94225750154 14.4947017381 12.4659501685
C 3.52861964244 14.4754036441 12.3577073653
C 5.59849751248 13.2623839713 12.7453836143
C 4.93906875044 12.0346886591 12.5939493810
C 3.59474190038 12.0104107355 12.1637960857
C 2.89586766061 13.2246637671 12.0684221991
C 6.94611912152 13.2582018755 13.3561687881
C 8.01061827123 12.5565803549 12.8033252623
C 9.25514208728 12.4894571308 13.4042516188
C 9.45503442388 13.1712344588 14.6006490919
C 8.42114110468 13.9041548335 15.1729940747
C 7.18066577936 13.9395725722 14.5562073332
C 5.60873361331 10.7366178432 12.8887448132
C 5.88152415448 10.3848699029 14.2069852658
C 6.42169249451 9.14281312921 14.5149741601
C 6.70576829764 8.23670264941 13.5028319753
C 6.44656104163 8.57948758430 12.1818092042
C 5.89796651284 9.81646305202 11.8793976536
C 2.92937753557 10.7109731927 11.8572820252
C 2.85494570181 9.65097639382 12.7660698796
C 2.19495897307 8.46470960544 12.4669845617
C 1.52283421818 8.32772145026 11.2676184918
C 1.54840620247 9.39171839865 10.3887040719
C 2.26602542826 10.5407618255 10.6476520212
C 1.52220384148 13.2362822293 11.5404761349
C 1.20383601710 14.1729390723 10.5633269963
C 16.7699616494 14.4711818953 10.2648893399
C 15.7580431622 13.6234813222 10.6986070430
C 16.0664545805 12.5399094202 11.5020326624
C 0.47893119339 12.4060599120 11.9798727656
C 15.3729283606 15.8460611699 13.0240836761
C 14.0280857218 15.8743906551 12.7500032346
C 15.4250707322 18.2198622449 12.8979676799

C	16.1311227552	17.0259008321	13.0459900083
C	14.0627330831	18.2894319275	12.5559568604
C	13.3491291216	19.5620280872	12.3594333910
C	11.9357149761	19.5470665848	12.4706621755
C	13.9855137351	20.8137204543	12.0878166485
C	13.2917276293	22.0193637636	12.2263539975
C	11.9550831338	21.9993288659	12.6621571484
C	11.2812286546	20.7774801751	12.7798415991
C	15.3481013590	20.8287554465	11.5406907111
C	15.6764382548	19.8883131275	10.5737692709
C	0.11068685522	19.6183152862	10.2545718401
C	1.11772953630	20.4688796313	10.7000925044
C	0.79782901096	21.5553958008	11.4913829441
C	16.3808057934	21.6824091173	11.9621637850
C	9.93903158754	20.7786877140	13.4014535892
C	9.71220803933	20.0358898742	14.5669168988
C	8.48171152232	20.0493470266	15.2033181191
C	7.44670132646	20.8182546487	14.6854432068
C	7.63993192089	21.5707851645	13.5316795116
C	8.87859859134	21.5360405673	12.9124389795
C	13.9365714143	23.3274495701	11.9269159274
C	14.2548883706	23.6317025654	10.6127859254
C	14.8805508311	24.8218547180	10.3036153046
C	15.2090864341	25.7513176382	11.2663233281
C	14.8738724708	25.4722898583	12.5839615804
C	14.2499594367	24.2741070673	12.9052583055
C	11.3254947801	23.3035968374	13.0093715856
C	11.0679559839	23.6152982537	14.3374004340
C	10.5210000079	24.8445259534	14.6867026974
C	10.2222346471	25.7786730196	13.7046720030
C	10.4875810802	25.4828392266	12.3718742078
C	11.0397744538	24.2597986997	12.0314861793
C	13.3571255903	17.0696627669	12.4551976792
C	11.9600800889	17.0782887619	12.0995450201
C	11.2314213228	18.2853980942	12.1959801977
C	11.3014579764	15.9134243544	11.6773824352
C	9.94268167074	15.8830864604	11.4782917782
C	9.85515634196	18.2241257089	11.9231323964
C	9.17483890276	17.0420418136	11.6489743655
H	1.03978381626	19.1425433791	13.2677674034
H	3.39396658515	19.0911797175	12.8416473408
H	0.90397017332	14.8966639104	12.9991189431
H	5.01010600500	19.0549464829	11.6018164688
H	7.38642837851	19.1131627731	11.2386237352
H	7.59506900064	14.9250116354	11.9404316742
F	7.81316512183	11.9507731854	11.5888013749
H	10.0497911653	11.9221142165	12.9356865384
H	10.4246850905	13.1327267991	15.0826530224

H	8.57741053807	14.4464405051	16.0977043828
H	6.36807849337	14.5097294696	14.9936388626
H	5.64454986297	11.0865887184	14.9988857776
H	6.61467141297	8.88760943298	15.5481852067
H	7.12896606060	7.26889708444	13.7398353054
H	6.66854569625	7.87841884801	11.3848677974
H	5.69469760025	10.0683591657	10.8448192951
H	3.32094271928	9.74471276346	13.7307511623
H	2.19633814024	7.65391786084	13.1844828044
H	0.97680434130	7.42957657006	11.0108459252
H	0.96213751100	9.33425872834	9.48426374871
H	2.24902640128	11.3550365593	9.93770484672
F	2.22201520068	14.8897750907	9.97445561502
H	16.5192207084	15.3930127459	9.75482740611
H	14.7337651279	13.8529051157	10.4465996847
H	15.2944495717	11.8381197269	11.7966356686
H	0.69184503692	11.6358002013	12.7057713021
H	15.8307014862	14.8875502909	13.2134712905
H	13.4804522119	14.9414790831	12.7597022740
H	15.9592229961	19.1410788833	13.0216454440
F	14.6682893422	19.1538228123	9.98264729469
H	0.35568754306	18.7095815403	9.72443972216
H	2.14177690502	20.2644120890	10.4226809349
H	1.56064753124	22.2691100966	11.7779925968
H	16.1631460423	22.4556496228	12.6813252364
H	10.5244517471	19.4375627448	14.9655560610
H	8.33616503353	19.4624715047	16.1018861623
H	6.48186990300	20.8296405238	15.1799478497
H	6.84473866647	22.1711608907	13.1067514010
F	9.06543425920	22.2439678859	11.7552054623
H	14.0179141285	22.9161857157	9.83383810928
H	15.1226937377	25.0433879938	9.27388961467
H	15.7030010440	26.6745525320	10.9922737418
H	15.0991055755	26.1934294584	13.3545253246
H	13.9996204247	24.0756180719	13.9373008356
H	11.3071444614	22.8935400209	15.1112011398
H	10.3320270616	25.0651723870	15.7276449520
H	9.78640890517	26.7318398464	13.9689183562
H	10.2575293341	26.2065452365	11.5983255094
H	11.2374762382	24.0377017257	10.9901731378
H	11.8655151189	15.0085756894	11.4963377979
H	9.48539541858	14.9546399157	11.1669696269
H	9.28257936752	19.1297308398	11.9608196683
Au	0.00000000000	0.00000000000	0.24315498000
Au	1.40653320000	2.61825445000	0.24315498000
Au	2.81306635000	0.00000000000	0.24315498000
Au	4.21959956000	2.61825445000	0.24315498000
Au	2.81306635000	5.23650889000	0.24315498000

Au	4.21959956000	7.85476334000	0.24315498000
Au	5.62613271000	5.23650889000	0.24315498000
Au	7.03266591000	7.85476334000	0.24315498000
Au	5.62613271000	10.4730177900	0.24315498000
Au	7.03266591000	13.0912722300	0.24315498000
Au	8.43919906000	10.4730177900	0.24315498000
Au	9.84573227000	13.0912722300	0.24315498000
Au	8.43919906000	15.7095266200	0.24315498000
Au	9.84573227000	18.3277810700	0.24315498000
Au	11.2522654200	15.7095266200	0.24315498000
Au	12.6587986200	18.3277810700	0.24315498000
Au	11.2522654200	20.9460355200	0.24315498000
Au	12.6587986200	23.5642899600	0.24315498000
Au	14.0653318300	20.9460355200	0.24315498000
Au	15.4718649800	23.5642899600	0.24315498000
Au	14.0653318300	26.1825444100	0.24315498000
Au	15.4718649800	28.8007988600	0.24315498000
Au	0.00000000000	26.1825444100	0.24315498000
Au	1.40653320000	28.8007988600	0.24315498000
Au	5.62613271000	0.00000000000	0.24315498000
Au	7.03266591000	2.61825445000	0.24315498000
Au	8.43919906000	0.00000000000	0.24315498000
Au	9.84573227000	2.61825445000	0.24315498000
Au	8.43919906000	5.23650889000	0.24315498000
Au	9.84573227000	7.85476334000	0.24315498000
Au	11.2522654200	5.23650889000	0.24315498000
Au	12.6587986200	7.85476334000	0.24315498000
Au	11.2522654200	10.4730177900	0.24315498000
Au	12.6587986200	13.0912722300	0.24315498000
Au	14.0653318300	10.4730177900	0.24315498000
Au	15.4718649800	13.0912722300	0.24315498000
Au	14.0653318300	15.7095266200	0.24315498000
Au	15.4718649800	18.3277810700	0.24315498000
Au	0.00000000000	15.7095266200	0.24315498000
Au	1.40653320000	18.3277810700	0.24315498000
Au	0.00000000000	20.9460355200	0.24315498000
Au	1.40653320000	23.5642899600	0.24315498000
Au	2.81306635000	20.9460355200	0.24315498000
Au	4.21959956000	23.5642899600	0.24315498000
Au	2.81306635000	26.1825444100	0.24315498000
Au	4.21959956000	28.8007988600	0.24315498000
Au	5.62613271000	26.1825444100	0.24315498000
Au	7.03266591000	28.8007988600	0.24315498000
Au	11.2522654200	0.00000000000	0.24315498000
Au	12.6587986200	2.61825445000	0.24315498000
Au	14.0653318300	0.00000000000	0.24315498000
Au	15.4718649800	2.61825445000	0.24315498000
Au	14.0653318300	5.23650889000	0.24315498000

Au	15.4718649800	7.85476334000	0.24315498000
Au	0.00000000000	5.23650889000	0.24315498000
Au	1.40653320000	7.85476334000	0.24315498000
Au	0.00000000000	10.4730177900	0.24315498000
Au	1.40653320000	13.0912722300	0.24315498000
Au	2.81306635000	10.4730177900	0.24315498000
Au	4.21959956000	13.0912722300	0.24315498000
Au	2.81306635000	15.7095266200	0.24315498000
Au	4.21959956000	18.3277810700	0.24315498000
Au	5.62613271000	15.7095266200	0.24315498000
Au	7.03266591000	18.3277810700	0.24315498000
Au	5.62613271000	20.9460355200	0.24315498000
Au	7.03266591000	23.5642899600	0.24315498000
Au	8.43919906000	20.9460355200	0.24315498000
Au	9.84573227000	23.5642899600	0.24315498000
Au	8.43919906000	26.1825444100	0.24315498000
Au	9.84573227000	28.8007988600	0.24315498000
Au	11.2522654200	26.1825444100	0.24315498000
Au	12.6587986200	28.8007988600	0.24315498000
Au	5.62613271000	3.49100591000	2.71166890000
Au	4.21959956000	0.87275146000	2.71166890000
Au	2.81306635000	3.49100591000	2.71166890000
Au	1.40653320000	0.87275146000	2.71166890000
Au	8.43919906000	8.72751480000	2.71166890000
Au	7.03266591000	6.10926036000	2.71166890000
Au	5.62613271000	8.72751480000	2.71166890000
Au	4.21959956000	6.10926036000	2.71166890000
Au	11.2522654200	13.9640237000	2.71166890000
Au	9.84573227000	11.3457692500	2.71166890000
Au	8.43919906000	13.9640237000	2.71166890000
Au	7.03266591000	11.3457692500	2.71166890000
Au	14.0653318300	19.2005325400	2.71166890000
Au	12.6587986200	16.5822780900	2.71166890000
Au	11.2522654200	19.2005325400	2.71166890000
Au	9.84573227000	16.5822780900	2.71166890000
Au	0.00000000000	24.4370414300	2.71166890000
Au	15.4718649800	21.8187869800	2.71166890000
Au	14.0653318300	24.4370414300	2.71166890000
Au	12.6587986200	21.8187869800	2.71166890000
Au	2.81306635000	29.6735503200	2.71166890000
Au	1.40653320000	27.0552958700	2.71166890000
Au	0.00000000000	29.6735503200	2.71166890000
Au	15.4718649800	27.0552958700	2.71166890000
Au	11.2522654200	3.49100591000	2.71166890000
Au	9.84573227000	0.87275146000	2.71166890000
Au	8.43919906000	3.49100591000	2.71166890000
Au	7.03266591000	0.87275146000	2.71166890000
Au	14.0653318300	8.72751480000	2.71166890000

Au	12.6587986200	6.10926036000	2.71166890000
Au	11.2522654200	8.72751480000	2.71166890000
Au	9.84573227000	6.10926036000	2.71166890000
Au	0.00000000000	13.9640237000	2.71166890000
Au	15.4718649800	11.3457692500	2.71166890000
Au	14.0653318300	13.9640237000	2.71166890000
Au	12.6587986200	11.3457692500	2.71166890000
Au	2.81306635000	19.2005325400	2.71166890000
Au	1.40653320000	16.5822780900	2.71166890000
Au	0.00000000000	19.2005325400	2.71166890000
Au	15.4718649800	16.5822780900	2.71166890000
Au	5.62613271000	24.4370414300	2.71166890000
Au	4.21959956000	21.8187869800	2.71166890000
Au	2.81306635000	24.4370414300	2.71166890000
Au	1.40653320000	21.8187869800	2.71166890000
Au	8.43919906000	29.6735503200	2.71166890000
Au	7.03266591000	27.0552958700	2.71166890000
Au	5.62613271000	29.6735503200	2.71166890000
Au	4.21959956000	27.0552958700	2.71166890000
Au	0.00000000000	3.49100591000	2.71166890000
Au	15.4718649800	0.87275146000	2.71166890000
Au	14.0653318300	3.49100591000	2.71166890000
Au	12.6587986200	0.87275146000	2.71166890000
Au	2.81306635000	8.72751480000	2.71166890000
Au	1.40653320000	6.10926036000	2.71166890000
Au	0.00000000000	8.72751480000	2.71166890000
Au	15.4718649800	6.10926036000	2.71166890000
Au	5.62613271000	13.9640237000	2.71166890000
Au	4.21959956000	11.3457692500	2.71166890000
Au	2.81306635000	13.9640237000	2.71166890000
Au	1.40653320000	11.3457692500	2.71166890000
Au	8.43919906000	19.2005325400	2.71166890000
Au	7.03266591000	16.5822780900	2.71166890000
Au	5.62613271000	19.2005325400	2.71166890000
Au	4.21959956000	16.5822780900	2.71166890000
Au	11.2522654200	24.4370414300	2.71166890000
Au	9.84573227000	21.8187869800	2.71166890000
Au	8.43919906000	24.4370414300	2.71166890000
Au	7.03266591000	21.8187869800	2.71166890000
Au	14.0653317700	29.6735503200	2.71166890000
Au	12.6587986200	27.0552958700	2.71166890000
Au	11.2522654200	29.6735503200	2.71166890000
Au	9.84573227000	27.0552958700	2.71166890000
Au	2.81306635000	1.74550293000	5.18018287000
Au	4.21959956000	4.36375743000	5.18018287000
Au	5.62613271000	1.74550293000	5.18018287000
Au	7.03266591000	4.36375743000	5.18018287000
Au	5.62613271000	6.98201182000	5.18018287000

Au	7.03266591000	9.60026627000	5.18018287000
Au	8.43919906000	6.98201182000	5.18018287000
Au	9.84573227000	9.60026627000	5.18018287000
Au	8.43919906000	12.2185207100	5.18018287000
Au	9.84573227000	14.8367751600	5.18018287000
Au	11.2522654200	12.2185207100	5.18018287000
Au	12.6587986200	14.8367751600	5.18018287000
Au	11.2522654200	17.4550296100	5.18018287000
Au	12.6587986200	20.0732840500	5.18018287000
Au	14.0653318300	17.4550296100	5.18018287000
Au	15.4718649800	20.0732840500	5.18018287000
Au	14.0653318300	22.6915385000	5.18018287000
Au	15.4718649800	25.3097928900	5.18018287000
Au	0.00000000000	22.6915385000	5.18018287000
Au	1.40653320000	25.3097928900	5.18018287000
Au	0.00000000000	27.9280473400	5.18018287000
Au	1.40653320000	30.5463017900	5.18018287000
Au	2.81306635000	27.9280473400	5.18018287000
Au	4.21959956000	30.5463017900	5.18018287000
Au	8.43919906000	1.74550293000	5.18018287000
Au	9.84573227000	4.36375743000	5.18018287000
Au	11.2522654200	1.74550293000	5.18018287000
Au	12.6587986200	4.36375743000	5.18018287000
Au	11.2522654200	6.98201182000	5.18018287000
Au	12.6587986200	9.60026627000	5.18018287000
Au	14.0653318300	6.98201182000	5.18018287000
Au	15.4718649800	9.60026627000	5.18018287000
Au	14.0653318300	12.2185207100	5.18018287000
Au	15.4718649800	14.8367751600	5.18018287000
Au	0.00000000000	12.2185207100	5.18018287000
Au	1.40653320000	14.8367751600	5.18018287000
Au	0.00000000000	17.4550296100	5.18018287000
Au	1.40653320000	20.0732840500	5.18018287000
Au	2.81306635000	17.4550296100	5.18018287000
Au	4.21959956000	20.0732840500	5.18018287000
Au	2.81306635000	22.6915385000	5.18018287000
Au	4.21959956000	25.3097928900	5.18018287000
Au	5.62613271000	22.6915385000	5.18018287000
Au	7.03266591000	25.3097928900	5.18018287000
Au	5.62613271000	27.9280473400	5.18018287000
Au	7.03266591000	30.5463017900	5.18018287000
Au	8.43919906000	27.9280473400	5.18018287000
Au	9.84573227000	30.5463017900	5.18018287000
Au	14.0653318300	1.74550293000	5.18018287000
Au	15.4718649800	4.36375743000	5.18018287000
Au	0.00000000000	1.74550293000	5.18018287000
Au	1.40653320000	4.36375743000	5.18018287000
Au	0.00000000000	6.98201182000	5.18018287000

Au	1.40653320000	9.60026627000	5.18018287000
Au	2.81306635000	6.98201182000	5.18018287000
Au	4.21959956000	9.60026627000	5.18018287000
Au	2.81306635000	12.2185207100	5.18018287000
Au	4.21959956000	14.8367751600	5.18018287000
Au	5.62613271000	12.2185207100	5.18018287000
Au	7.03266591000	14.8367751600	5.18018287000
Au	5.62613271000	17.4550296100	5.18018287000
Au	7.03266591000	20.0732840500	5.18018287000
Au	8.43919906000	17.4550296100	5.18018287000
Au	9.84573227000	20.0732840500	5.18018287000
Au	8.43919906000	22.6915385000	5.18018287000
Au	9.84573227000	25.3097928900	5.18018287000
Au	11.2522654200	22.6915385000	5.18018287000
Au	12.6587986200	25.3097928900	5.18018287000
Au	11.2522654200	27.9280473400	5.18018287000
Au	12.6587986200	30.5463017900	5.18018287000
Au	14.0653317700	27.9280473400	5.18018287000
Au	15.4718649800	30.5463017900	5.18018287000
Au	5.62986566971	31.4058181029	9.00747338934
Au	7.04359001563	2.57681159278	8.92439207855
Au	5.64398954451	5.12418670998	8.76588871709
Au	4.23505275944	2.54566976607	9.09508095309
Au	8.45420379311	5.18069087781	8.86369341361
Au	9.85285497202	7.76866603812	9.08403698274
Au	8.51939671217	10.3553005797	8.61999111753
Au	7.08261923955	7.73700272421	8.39289772226
Au	11.2723618172	10.3523566656	9.17119853034
Au	12.6384240706	12.8941316182	8.63691970412
Au	11.2945707978	15.5242404800	8.22238125001
Au	9.85347926978	12.9627240303	8.69062839795
Au	14.0665172355	15.5226062625	7.94963086921
Au	15.4384151761	18.2242759778	7.57818925798
Au	13.9854708264	21.0248697723	7.69465704005
Au	12.5726848823	18.2075738115	7.88987046209
Au	0.11553055270	21.0865032362	7.70887045667
Au	1.49128687996	23.8204929892	8.34454261627
Au	0.16866845926	26.5249034545	7.85930427924
Au	15.6192873658	23.6112872161	7.56981291743
Au	2.78039940820	26.3154803804	9.08295416501
Au	4.22224368912	28.8491570255	9.26244180425
Au	2.82553990563	0.00204123805	9.25175178787
Au	1.40922728212	28.8930636386	9.09385887763
Au	11.2421707168	0.05447942073	9.05749579204
Au	12.6560260251	2.61675387036	9.17316867258
Au	11.2542298431	5.19199119621	9.15867731625
Au	9.85348394075	2.61414168591	8.93462176712
Au	14.0691247448	5.15831539901	9.21010037855

Au	15.4466390056	7.62721874076	8.54287099459
Au	14.0503319035	10.2689408764	8.75522397000
Au	12.6838231430	7.75049844453	9.21865867229
Au	16.6944108488	10.1999022454	7.71640255332
Au	1.38844872446	12.8712327237	7.66404049857
Au	0.06522506659	15.7481319054	7.57208065828
Au	15.3312198483	12.7848990681	7.80640046250
Au	3.02100682246	15.9393013556	7.64877563583
Au	4.26461615710	18.5811739527	8.34540872164
Au	2.97972907477	21.2898106653	7.96404041118
Au	1.53834537956	18.4307003065	7.69589786466
Au	5.63870299419	21.1417159752	8.84157759685
Au	7.02499528837	23.6870134403	9.04035597519
Au	5.62381076646	26.2780271741	9.31838727133
Au	4.20469403059	23.7336906625	9.14604530609
Au	8.41822683042	26.2681425853	8.81748876103
Au	9.83639512600	28.8799408154	8.88448692096
Au	8.43543532663	0.01491485316	8.88580800774
Au	7.02449557776	28.8383362644	9.01911641652
Au	0.00391390742	0.02572105110	9.28192671297
Au	1.41722843372	2.55631707844	9.27715968664
Au	16.8767779898	5.08473008924	9.00008782655
Au	15.4675075815	2.58348448082	9.32605647787
Au	2.83069050785	5.06004638284	8.79738721081
Au	4.24976034978	7.60780563729	8.04551328213
Au	2.96080423804	10.3237634689	7.64642249648
Au	1.36169426183	7.35178380235	7.77031478738
Au	5.77628753838	10.3883684460	8.00824492933
Au	7.07153084708	13.0616588163	8.34406744057
Au	5.74738656228	15.7897304428	8.20431835646
Au	4.28321306867	13.1218319304	7.92401529126
Au	8.51799970722	15.6461206563	8.06421685341
Au	9.80851416073	18.3390853904	8.16024810687
Au	8.41993417615	21.0408478817	8.53157324476
Au	7.03066718132	18.4771256128	8.17494902738
Au	11.2045275523	20.9671625480	8.20233799314
Au	12.5191953421	23.7115410303	7.70940837366
Au	11.1940819581	26.3715387434	8.20873845535
Au	9.74288771083	23.6710021516	8.28197643078
Au	14.0112007313	26.6298588797	7.73304720359
Au	15.4862835524	28.9523403237	8.75043217850
Au	14.0629092727	0.05236197008	9.19433988749
Au	12.6475422042	28.9424590438	8.85600012299
